# Global, regional, and national burden of laryngeal cancer attributable to smoking, 1990–2021, and projections to 2036: a systematic analysis of the Global Burden of Disease study 2021

**DOI:** 10.3389/fpubh.2025.1583045

**Published:** 2025-05-09

**Authors:** Liangwen Shi, Zhixing Kuang, Jiannan Tu, Taiqin Wang, Tao Liu, Jingbo Liu, Jianzhi Liu

**Affiliations:** ^1^Department of Otorhinolaryngology, Fujian Medical University Union Hospital, Fuzhou, China; ^2^Department of Radiotherapy, Fujian Medical University Union Hospital, Fuzhou, China; ^3^Department of Medical Oncology, Fudan University Shanghai Cancer Center Xiamen Hospital, Xiamen, China

**Keywords:** laryngeal cancer attributable to smoking (LCAS), burden of disease, temporal trends, projection, disparities

## Abstract

**Background:**

Laryngeal cancer, predominantly linked to smoking, persists as a formidable global health challenge. Acquiring a deeper understanding of laryngeal cancer attributable to smoking (LCAS) burden and trends is essential for crafting nuanced and impactful prevention and intervention strategies.

**Methods:**

The Disability-Adjusted Life Years (DALYs), mortality, and corresponding Age-Standardized DALY rate (ASDR) and Age-Standardized Mortality Rate (ASMR) were systematically evaluated using the GBD 2021 data. Temporal trends were characterized through Joinpoint regression analysis, while the influence of socioeconomic factors on disease distribution and the associated disparities was probed through correlation analysis. Decomposition analysis was employed to identify the key factors driving changes in DALY and mortality burdens, and the Bayesian Age-Period-Cohort (BAPC) model was used to project future trends.

**Results:**

Over the past 30 years, the global burden of LCAS, as measured by DALYs and the number of deaths, has remained relatively stable with a modest increase of approximately 20.8 and 11.53%, respectively. However, both ASDR and ASMR have shown a decline, from 44.42 to 23.38 and from 1.61 to 0.89, respectively. Regionally, the burden is predominantly concentrated in Asia, Europe, as well as North Africa and the Middle East. In all regions, both ASDRs and ASMRs are on a downward trajectory, with the rate of decline being more pronounced as socio-economic indices (SDI) rise. Notably, Australasia, High-income Asia Pacific, and Western Europe exhibit the largest annual reductions in ASDRs and ASMRs. Population aging has been identified as the primary driver of DALYs and mortality burden in High-income Asia Pacific, while population growth plays a more significant role in other regions. In the next 15 years, both ASDR and ASMR are expected to continue their decline.

**Conclusion:**

Despite progress in reducing LCAS rates over 30 years, regional disparities persist, strengthened tobacco control measures are essential to further alleviate the LCAS burden and reduce smoking-related mortality.

## Introduction

Laryngeal cancer is characterized as a malignant neoplasm located in the larynx and represents one of the most prevalent malignancies within the head and neck region, constituting approximately 20% of all newly diagnosed cancers worldwide (1, 2). The incidence of laryngeal cancer demonstrates notable regional variability, with areas such as Central Europe, Eastern Europe, Cuba, Spain, and Uruguay exhibiting relatively high rates (3). While the precise etiology of laryngeal cancer remains unclear, evidence suggests an association with the synergistic effects of multiple factors, including tobacco use, alcohol consumption, air pollution, and occupational exposures (4). Tobacco smoking exhibits a robust and well—established correlation with the onset of laryngeal cancer. Multiple comprehensive studies have indicated that smoking can increase the likelihood of developing laryngeal cancer by approximately 10 to 15 times. For individuals with the most severe smoking addiction, the risk can be as high as 30 times (5). Benzo[a]pyrene, a constituent of tobacco tar generated during tobacco combustion, possesses potent carcinogenic properties. It can instigate mucosal edema, congestion, epithelial hyperplasia, and squamous metaplasia. Additionally, it inhibits ciliary movement, thus fostering the process of carcinogenesis (6). The pathogenic mechanism of smoking—induced laryngeal cancer potentially involves the irreversible mutation of crucial genes. This is a consequence of exposure to carcinogens within tobacco smoke, which disrupts normal growth regulatory mechanisms. Moreover, smoking may promote carcinogenesis via mechanisms like modifying oxidative stress levels, compromising immune function, and modulating cellular inflammatory responses (7). In 2020, the global number of newly diagnosed laryngeal cancer cases was 184,615, with 99,840 deaths attributed to the disease, accounting for over 1% of total cancer-related mortality (3). Compelling reports further indicate that a staggering 63.5% of these laryngeal—cancer—related deaths on a global scale can be directly attributed to smoking (8).

The marked divergence in smoking behaviors and the stringency of tobacco control policies across various regions has led to substantial disparities in the incidence and mortality of cancer attributable to smoking (9). In developed nations with robust smoking control strategies, both the incidence and mortality rates of laryngeal cancer have demonstrated a downward trajectory (10). These comprehensive measures, including strict tobacco taxation, public awareness campaigns, and restrictions on tobacco advertising, have effectively curbed smoking prevalence and subsequently reduced the burden of laryngeal cancer (11). Conversely, within select developing nations, exemplified by Sri Lanka, where tobacco control endeavors are inadequately stringent, both the incidence and mortality of laryngeal cancer exhibit an upward trajectory (12). The paucity of comprehensive tobacco control policies, compounded by a high prevalence of smoking, significantly contributes to this concerning trend. This scenario not only underscores the urgent imperative for the implementation of more efficacious tobacco control measures in these regions but also highlights the far—reaching global health implications stemming from inconsistent tobacco regulation.

The initial presentations of laryngeal cancer are often subtle and insidious, frequently escaping patients’ awareness. This phenomenon culminates in a significant proportion of cases being diagnosed at an advanced stage (13). Management of advanced laryngeal cancer typically entails multimodal therapeutic approaches, integrating surgical resection, radiotherapy, and chemotherapy. These interventions not only incur substantial financial costs but also demand protracted treatment courses, thereby imposing a considerable economic strain on patients and their families (14). Furthermore, the prognosis for advanced laryngeal cancer remains suboptimal. Patients are confronted with a relatively high probability of recurrence and the emergence of secondary primary neoplasms (15). Consequently, comprehensive epidemiological investigations into laryngeal cancer are of paramount importance. Such research is instrumental in refining early detection techniques, fortifying preventive measures, and optimizing treatment regimens. Ultimately, these efforts can contribute to a substantial reduction in the disease burden and an enhancement of patients’ quality of life.

While the association between smoking and laryngeal cancer has been well-documented, large-scale epidemiological studies examining global disparities through theoretical frameworks remain limited. This study employs an integrated analytical approach grounded in population health theory to address this gap. The Global Burden of Disease (GBD) framework informs our quantification of health loss through DALYs and Mortality identifying high-priority regions, while the Social Determinants of Health perspective elucidates observed socioeconomic gradients in disease burden. Furthermore, the Socio-Ecological Model enables systematic examination of risk factors across individual, community, and policy levels (16). Leveraging the latest data from the 2021 GBD study, this research conducts an in—depth assessment of the changing trends in disability—adjusted life years (DALYs), mortality counts, age—standardized disability—adjusted life years (ASDRs), and age—standardized mortality rates (ASMR) caused by smoking—related laryngeal cancer across 21 regions and 204 countries from 1990 to 2021. Furthermore, this study delves deep into the driving factors behind the changes in disease burden, explores its correlation with the Socio—demographic Index (SDI), and forecasts the burden status over the next 15 years. The results of this study offer crucial data that can guide the formulation of global tobacco control policies, the implementation of targeted interventions for high—risk groups, and the improvement of disease prevention and control strategies. Consequently, it plays a vital role in alleviating the global burden of laryngeal cancer.

## Method

### Study design, data source, and variables

This study is a retrospective time-trend analysis, classified as a descriptive and predictive epidemiological study, utilizing secondary data from the Global Burden of Disease (GBD) 2021 database, accessed via the Institute for Health Metrics and Evaluation (IHME) website.^1^ It evaluates the burden of smoking-related laryngeal cancer (LCAS) across 204 countries and 21 regions from 1990 to 2021, focusing on DALYs, mortality, ASDR, and ASMR, alongside associations with the Socio-demographic Index (SDI) and 15-year burden forecasts. Data were extracted from the GBD Results Tool for laryngeal cancer (ICD-10 code C32-C32.9), covering DALYs, mortality counts, ASDR, and ASMR, stratified by sex (male, female), age groups (30–95 + years, 5-year intervals), regions (e.g., South Asia, Central Europe), and countries (e.g., China, India). Smoking-related burden was estimated using GBD’s comparative risk assessment, attributing risk based on smoking exposure. GBD’s standardized protocols, including ICD-10 coding and age-weighting, ensured data consistency, with missing values handled via GBD imputation methods. Over the past 30 years, the GBD study has refined global estimates for 371 diseases and injuries. GBD 2021, following GBD 2019, offers an up-to-date evaluation of global health trends, including disruptions from the COVID-19 pandemic. The primary outcome variables were defined as follows: DALYs: Disability-adjusted life years, combining years of life lost (YLLs) and years lived with disability (YLDs), calculated as DALYs = YLLs + YLDs. YLLs were derived from mortality and standard life expectancy; YLDs used GBD disability weights Mortality: Annual LCAS deaths. ASDR: Age-standardized death rate per 100,000. ASMR: Age-standardized mortality rate per 100,000. Analytical dimensions included sex (male, female), region (21 GBD regions), country (204 countries), and SDI (low to high quintiles). SDI served as a covariate for socioeconomic effects, while sex and region enabled disparity analyses.

### Joinpoint regression analysis and decomposition analysis

Temporal trends in ASDRs and ASMRs attributable to LCAS were analyzed through Joinpoint regression across global, regional, and national levels from 1990 to 2021. Segmented regression models were optimized via grid search to minimize the mean squared error (MSE) and further validated by Monte Carlo permutation tests, this approach is commonly employed in authoritative studies on the global epidemiology of other cancers (17). To ensure comparability across a wide array of regions and countries over the extended time span, the optimal number of joinpoints was determined to be five. Moreover, to mitigate the influence of short-term fluctuations and ensure robustness of the results, the Average Annual Percent Change (AAPC) was employed as the primary metric for assessing trend variations across the different regions, countries, and territories. AAPC values were calculated using the formula:


$$\text{AAPC}=\{\exp(\sum {\tilde{w}}_{i}b_{i})-1\}\times 100$$

Where ${\tilde{w}}_{i}=w_{i}/\sum w_{j}$ denotes the normalized weights, and $b_{i}$ refers to the annual percent change (APC) estimate for each segment, which is obtained from the Joinpoint model fit.

The variations in mortality and DALY burden between 1990 and 2021 were dissected by applying the Das Gupta decomposition method to quantify the individual contributions of population aging, growth, and epidemiological changes (18). This analysis provides a comprehensive understanding of how each factor independently influences the overall changes in disease burden, thus highlighting the major determinants at play. Such insights are crucial for developing precise, evidence-informed strategies to tackle the root causes of these shifts.

### BAPC model projection

The Bayesian Age-Period-Cohort (BAPC) model was employed to forecast the global future burden of LCAS, including DALYs, mortality, ASDR, and ASMR, over the next 15 years. By leveraging the Integrated Nested Laplace Approximation (INLA) methodology, the Bayesian Age-Period-Cohort (BAPC) model incorporates temporal trends across age, period, and cohort dimensions. This model employs Bayesian hierarchical frameworks to effectively mitigate uncertainty, thereby enhancing the precision of its estimates. The BAPC model’s notable strengths lie in its flexibility, which circumvents stringent parametric assumptions, its capacity to integrate prior knowledge, and its ability to generate probabilistic forecasts. These attributes enable the model to accommodate data heterogeneity and yield reliable long-term projections. Comparative evaluations consistently demonstrate the superior predictive performance of the BAPC model when contrasted with conventional methods, such as linear power and APC models (19).

### Statistics

All statistical analyses supported result comparisons, including trends, disparities, and projections, with significance at *p* < 0.05. All age-standardized rates were per 100,000 population with 95% uncertainty intervals (UI). Spearman’s rank correlation tested associations between SDI and burden metrics. All analyses were performed using appropriate statistical models, with statistical significance determined at *p* < 0.05. Analyses used the World Health Organization’s Health Equity Assessment Toolkit and R software (version 4.4.1).

## Results

### The global and regional burden of LCAS in 2021

**Figure 1:**
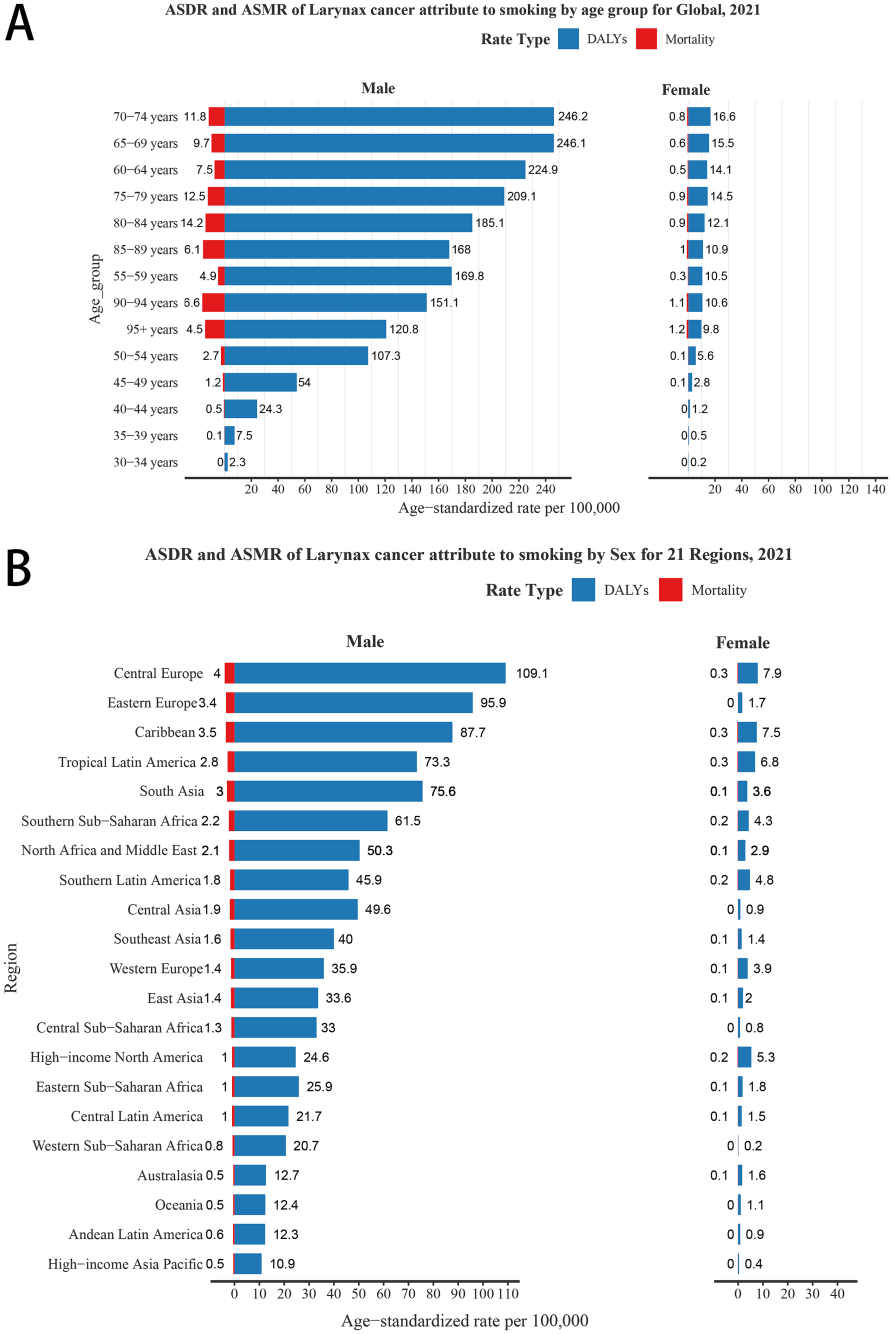
ASDR and ASMR of laryngeal cancer attributable to smoking (LCAS) by sex for global and 21 GBD regions. **(A)** By age group and sex globally in 2021. **(B)** By sex across 21 GBD regions in 2021. ASDR: Age-Standardized Disability Rate (per 100,000); ASMR: Age-Standardized Mortality Rate (per 100,000).

Globally, the LCAS burden in 2021 amounted to 2,061,113 DALYs (95% UI: 1,838,588–2,281,339) and 72,740 deaths (95% UI: 64,992–80,488), with a corresponding age-standardized death rate (ASDR) of 23.38 per 10,000 population (95% UI: 20.84–25.89) and an age-standardized mortality ratio (ASMR) of 0.89 per 10,000 population (95% UI: 0.80–0.99) (Table 1). By gender, the burden is heavily concentrated in males, with female ASDR and ASMR values less than one-tenth of those for males (Figure 1A). This disparity is evident across all GBD regions. When stratified by age group, the highest ASDR for both males and females is observed in the 70–74, 65–69, 60–64, and 75–79. In terms of ASMR, the highest values for both genders are found in the 80–84 and 75–79 age groups. Notably, the lowest ASDR and ASMR for both males and females are recorded in the 30–34 age group (Figure 1A).

**Table 1:** The mortality and DALYs of laryngeal cancer attribute to smoking, and related AAPCs from 1990 to 2021 at the global level and different regions.

Characteristics	Mortality			DALYs		
	Counts, 2021	Age-standardized rate per 100 000,2021	AAPC 1990–2021	Counts, 2021	Age-standardized rate per 100 000,2021	AAPC, 1990–2021
Global	77951 (69,554, 86,284)	0.89 (0.8, 0.99)	−1.90 (−2.00–−1.80)	2,061,113 (1,838,588, 2,281,339)	23.38 (20.84, 25.89)	−2.00 (−2.10–−1.90)
Sex						
Males	72740 (64,992, 80,488)	1.8 (1.6, 1.99)	-1.90 (−2.0- -1.90)	1,930,971 (1,728,673, 2,137,743)	45.86 (41.08,50.77)	−2.10 (−2.2–−2.0)
Females	5211 (4,212, 6,357)	0.11 (0.09, 0.14)	−1.80 (−2.0–−1.60)	13,0142 (106,768, 157,603)	2.83 (2.32, 3.42)	−1.9 (−2.1–−1.7)
GBD region						
Andean Latin America	162 (121, 211)	0.56 (0.41, 0.73)	−2.30 (−2.90–−1.80)	3,741 (2,808, 4,883)	6.33 (4.74, 8.27)	−2.40 (−3.00–−1.90)
Australasia	147 (117, 177)	0.51 (0.41, 0.61)	−3.70 (−4.50–−3.00)	3,457 (2,823, 4,064)	6.95 (5.67, 8.18)	−3.90 (−4.70–−3.20)
Caribbean	971 (800, 1,157)	3.47 (2.85, 4.14)	−0.40 (−1.10–0.30)	24,805 (20,503, 29,668)	45.6 (37.75, 54.48)	−0.30 (−1.00–0.40)
Central Asia	708 (621, 802)	1.88 (1.66, 2.13)	−2.80 (−3.60–−2.00)	20,604 (18,020, 23,339)	22.58 (19.73, 25.61)	−3.20 (−4.00–−2.40)
Central Europe	4078 (3,600, 4,529)	3.95 (3.49, 4.39)	−1.50 (−1.60–−1.30)	109,911 (97,442, 121,846)	55.03 (48.85, 61.02)	−1.70 (−1.80–−1.50)
Central Latin America	1152 (945, 1,374)	0.96 (0.79, 1.14)	−3.30 (−4.00–−2.70)	27,309 (22,234, 32,749)	10.77 (8.79, 12.9)	−3.40 (−4.00–−2.80)
Central Sub-Saharan Africa	325 (239, 433)	1.27 (0.94, 1.67)	−0.80 (−1.00–−0.60)	9,847 (7,174, 13,202)	15.52 (11.45, 20.62)	−0.80 (−1.00–−0.60)
East Asia	15666 (11,981, 20,277)	1.42 (1.08, 1.84)	−1.70 (−2.00–−1.50)	392,266 (29,4314, 508,422)	17.23 (12.99, 22.28)	−1.90 (−2.10–−1.60)
Eastern Europe	4824 (4,180, 5,446)	3.37 (2.91, 3.81)	−2.10 (−2.80–−1.50)	139,249 (120,265, 157,679)	41.31 (35.71, 46.82)	−2.40 (−3.00–−1.70)
Eastern Sub-Saharan Africa	848 (640, 1,110)	0.98 (0.74, 1.26)	−1.40 (−1.50–−1.30)	25,248 (18,805, 33,483)	13.35 (10.03, 17.47)	−1.40 (−1.40–−1.30)
High-income Asia Pacific	1178 (998, 1362)	0.5 (0.43, 0.58)	−3.50 (−3.90–−3.00)	23,284 (19,811, 27,147)	5.26 (4.49, 6.19)	−3.70 (−4.00–−3.40)
High-income North America	3577 (3,147, 3,985)	0.96 (0.84, 1.06)	−2.30 (−2.60–−1.90)	90,593 (81,178, 99,608)	14.41 (12.97, 15.81)	−2.40 (−2.80–−2.10)
North Africa and Middle East	4785 (4,067, 5,574)	2.08 (1.77, 2.4)	−1.40 (−1.50–−1.40)	130,351 (110,438, 152,839)	26.94 (22.92, 31.42)	−1.60 (−1.70–−1.50)
Oceania	20 (14, 26)	0.52 (0.39, 0.72)	−0.80 (−1.10–−0.60)	557 (405, 778)	6.91 (5.06, 9.38)	−0.80 (−1.10–−0.50)
South Asia	22275 (18,553, 26,029)	2.96 (2.48, 3.45)	−1.30 (−1.60–−1.00)	609,830 (510,032, 716,751)	39.06 (32.66, 45.79)	−1.40 (−1.60–−1.20)
Southeast Asia	5012 (4,288, 5,878)	1.64 (1.4, 1.9)	−0.80 (−0.90–−0.60)	135,943 (114,899, 161,411)	19.36 (16.47, 22.87)	−0.80 (−0.90–−0.60)
Southern Latin America	777 (666, 888)	1.78 (1.52, 2.05)	−2.90 (−3.30–−2.50)	20,084 (17,536, 22,805)	23.66 (20.7, 26.9)	−3.20 (−3.60–−2.70)
Southern Sub-Saharan Africa	617 (516, 720)	2.24 (1.89, 2.59)	−1.10 (−1.60–−0.60)	18,540 (15,458, 21,606)	29.04 (24.22, 33.87)	−1.10 (−1.50–−0.60)
Tropical Latin America	3633 (3,182, 4,100)	2.77 (2.42, 3.12)	−1.60 (−1.90–−1.20)	99,359 (87,068, 111,467)	37.45 (32.79, 42.04)	−1.60 (−2.00–−1.30)
Western Europe	6459 (5,671, 7,201)	1.4 (1.25, 1.56)	−3.20 (−3.50–−2.80)	154,765 (139,543, 170,773)	19.1 (17.36, 20.98)	−3.40 (−3.80–−3.00)
Western Sub-Saharan Africa	737 (558, 900)	0.79 (0.61, 0.97)	−0.50 (−0.50–−0.40)	21,369 (15,894, 26,290)	9.81 (7.4, 12)	−0.50 (−0.60–−0.50)

Regionally, South Asia, East Asia, and Western Europe rank among the top three for both DALYs and deaths due to LCAS in 2021. For ASDR, Central Europe leads at 55.03 per 10,000 population (95% UI: 48.85–61.02), followed by the Caribbean at 45.60 (95% UI: 37.75–54.48) and Eastern Europe at 41.31 (95% UI: 35.71–46.82). The highest ASMR are observed in Central Europe at 1.93 per 10,000 (95% UI: 1.70–2.14), the Caribbean at 1.79 (95% UI: 1.47–2.13), and South Asia at 1.51 (95% UI: 1.26–1.76). In contrast, the lowest ASMRs are recorded in Australasia at 0.28 (95% UI: 0.21–0.37) and High-income Asia Pacific at 0.27 (95% UI: 0.22–0.33), reflecting a significantly lower burden in these regions (Table 1). When disaggregated by gender, Central Europe, Western Europe, and the Caribbean leading globally in male ASDR (109.1, 95.9, and 87.7 per 10,000 population, respectively) and ASMR (4.0, 3.4, and 3.5 per 10,000, respectively). For females, Central Europe, the Caribbean, and Tropical Latin America report the highest ASDRs (7.9, 7.5, and 6.8 per 10,000, respectively), each with a consistent ASMR of 0.3. Conversely, regions with the lowest male burden, including High-income Asia Pacific, Andean Latin America, Oceania, and Australasia, show significantly reduced ASDR and ASMR (Figure 1B).

### The national burden of LCAS in 2021

**Figure 2:**
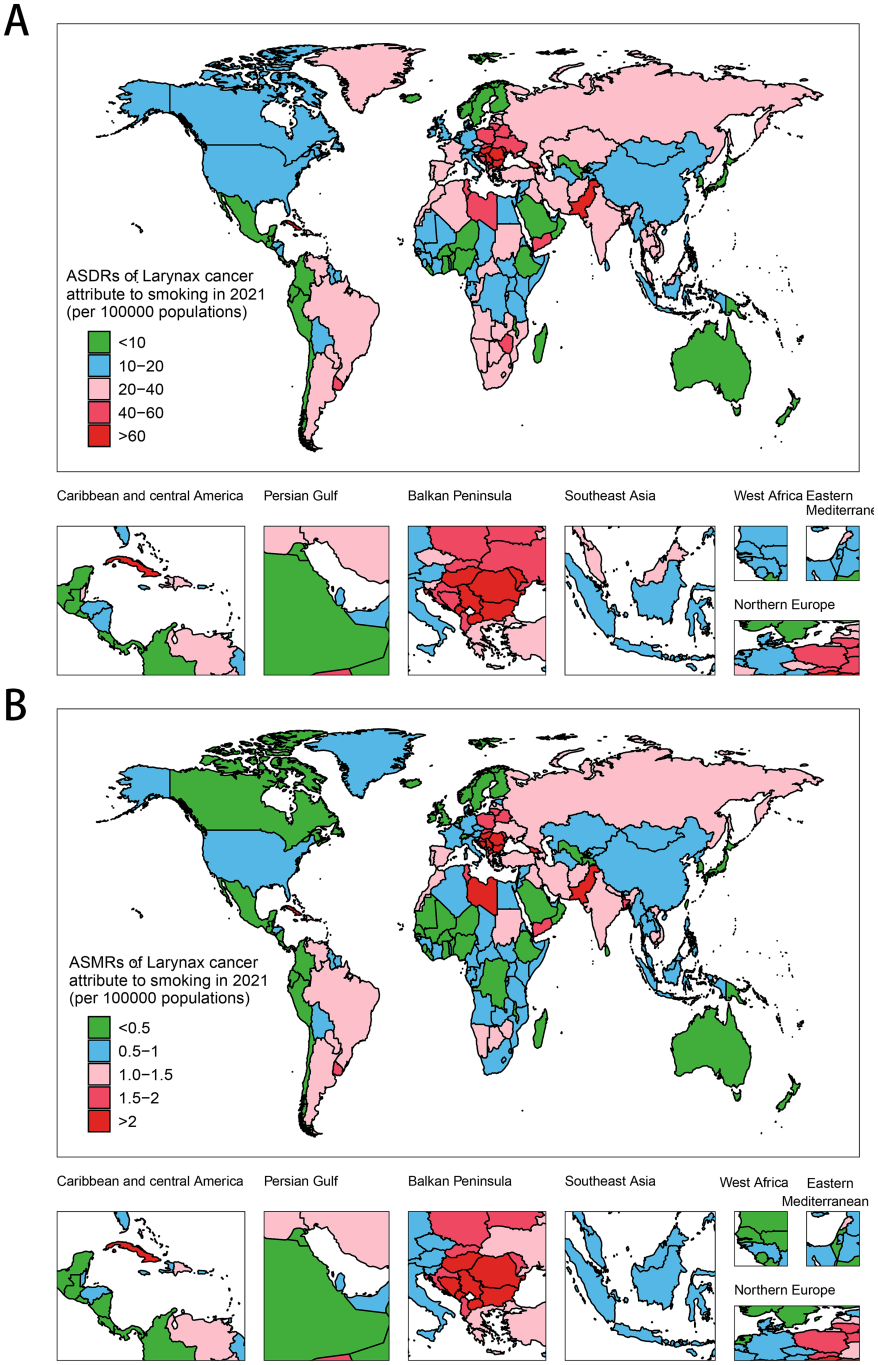
Global distribution of ASMRs and ASDRs of LCAS in 2021. **(A)** ASDR. **(B)** ASMR.

When it comes to specific countries and territories, India leads in DALYs (444,574; 360,630–531,702), followed by China (381,556; 284,584–497,331), Brazil (97,517; 95% UI: 85,520–109,503), Russia, and the US. Montenegro has the highest ASDR (103.93; 77.66–141.07), ahead of Cuba, Pakistan, Seychelles, and Bulgaria, while Guam (3.92; 3.21–4.82) and Japan (4.07; 3.65–4.46) show the lowest rates (Figure 2A, Supplementary Table S1). With regard to mortality burden, India records the highest number of deaths, estimated at 16,321 (95% UI: 13,391–19,365), followed by China with 15,274 (95% UI: 11,616–19,867), Pakistan at 3,576 (95% UI: 2,513–4,837), Brazil with 3,564 (95% UI: 3,124–4,020), and the United States reporting 3,279 (95% UI: 2,894–3,638). In terms of ASMRs, Montenegro emerges as the highest at 3.68 (95% UI: 2.76–4.97), with Cuba (3.41; 95% UI: 2.76–4.14), Seychelles (3.03; 95% UI: 2.41–3.7), Pakistan (3.00; 95% UI: 2.12–4.02), and North Macedonia (2.62; 95% UI: 1.92–3.45) following closely (Figure 2B, Supplementary Table S1). In the five regions with the highest LCAS DALYs, Pakistan led in South Asia with an ASDR of 76.79 per 10,000 population (95% UI: 54.06–104.02), while China recorded the highest in East Asia at 17.34 (95% UI: 12.97–22.59). Monaco topped Western Europe with 68.43 (95% UI: 50.10–97.31), the Republic of Moldova ranked highest in Eastern Europe at 60.33 (95% UI: 52.41–68.59), and Seychelles led Southeast Asia with 76.01 (95% UI: 60.17–93.80) (Supplementary Figure S1A, Supplementary Table S1). Notably, the ASMRs in these countries closely mirrored their ASDR rankings, reflecting a consistent burden pattern across regions. Similarly, in Central Asia, Eastern Africa, and high-income East Asia, Georgia, Libya, and Brunei Darussalam exhibited the highest ASDRs and ASMRs in their respective regions, while Uzbekistan, Kuwait, and Japan recorded the lowest rates (Supplementary Figure S1B, Supplementary Table S1).

### Trends in the burden of LCAS from 1990 to 2021

The global burden of LCAS increased by 11.5% from 1,848,004 DALYs (95% UI: 1,688,370–1,995,684) in 1990 to 2,061,113 DALYs (95% UI: 1,838,588–2,281,339) in 2021. The number of deaths has also increased from 64,528 (95% UI: 58,868–69,741) in 1990 to 77,951 (95% UI: 69,554–86,284) in 2021(Table 1, Supplementary Table S2). Regionally, from 1990 to 2021, the burden of LCAS has experienced significant shifts across various regions. In South Asia, DALYs rose from 386,047 (95% UI: 318,977–459,765) in 1990 to 609,830 (95% UI: 510,032–716,751) in 2021. East Asia saw an increase from 282,986 (95% UI: 228,635–336,596) in 1990 to 392,266 (95% UI: 294,314–508,422) in 2021. Western Europe experienced a decrease from 292,190 (95% UI: 272,561–311,381) in 1990 to 154,765 (95% UI: 139,543–170,773) in 2021. Eastern Europe and Central Europe also showed reductions in their burden: Eastern Europe fell from 250,156 (95% UI: 236,016–264,173) in 1990 to 139,249 (95% UI: 120,265–157,679) in 2021, and Central Europe dropped from 139,647 (95% UI: 130,170–149,362) in 1990 to 109,911 (95% UI: 97,442–121,846) in 2021(Table 1, Supplementary Table S2).

**Figure 3:**
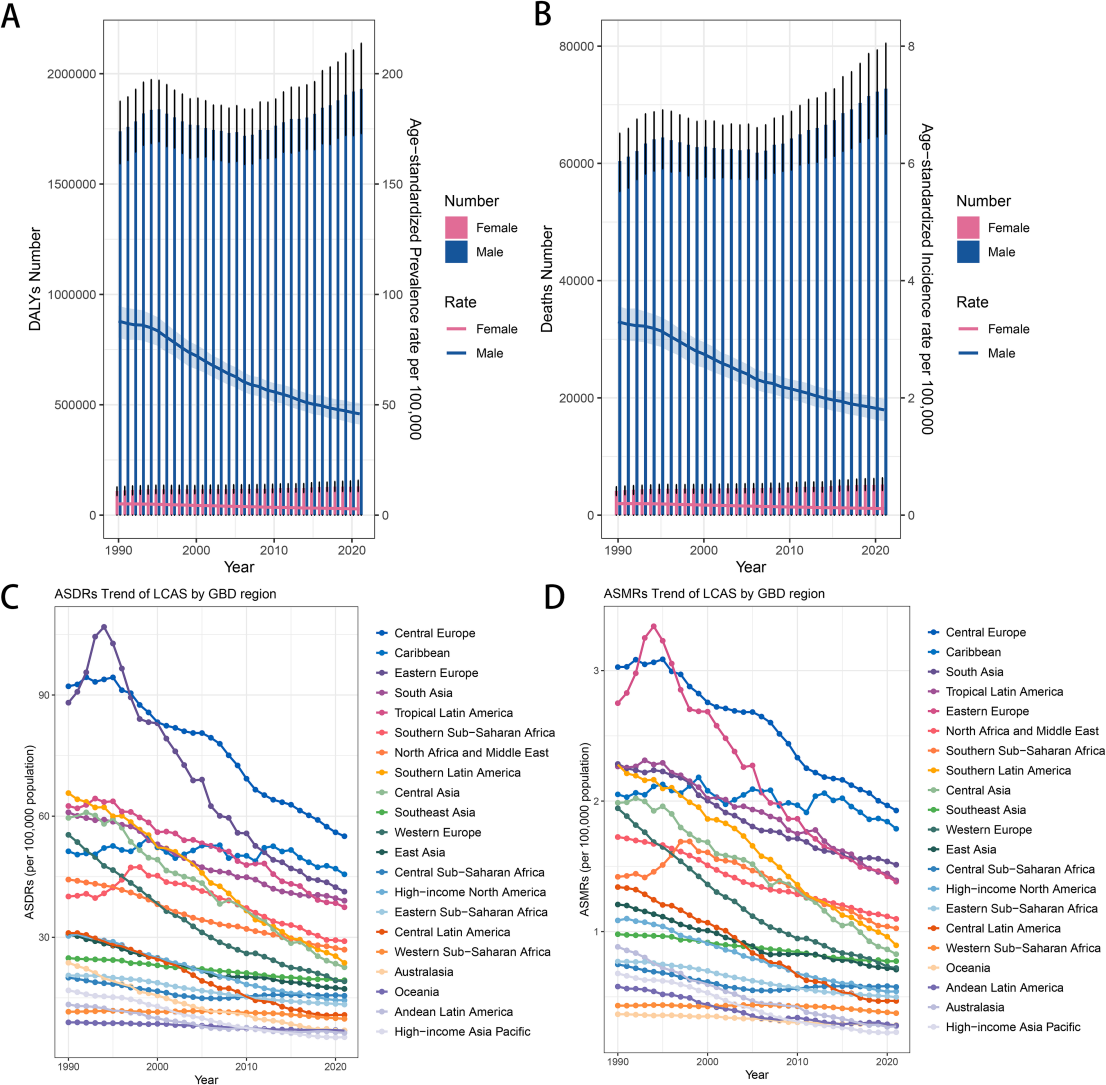
Trend changes of LCAS ASDRs and ASMRs in global and world regions, 1990–2021. **(A)** Global trends in DALYs number and ASDRs by sex. **(B)** Global trends in death number and ASMRs by sex. **(C)** ASDR trends of LCAS by GBD region. **(D)** ASMR trends of LCAS by GBD region.

Between 1990 and 2021, ASDRs and ASMRs for LCAS significantly decreased across several regions. In Australasia, ASDR dropped from 23.71 (95% UI: 21.15–26.8) to 6.33 (95% UI: 4.74–8.27), with an AAPC of −3.90 (95% CI: −4.70 to −3.20). High-income Asia Pacific decreased from 16.85 (95% UI: 14.43–19.13) to 5.26 (95% UI: 4.49–6.19), with an AAPC of −3.70 (95% CI: −4.00 to −3.40). Central Latin America saw a reduction from 31.06 (95% UI: 28.11–33.86) to 10.77 (95% UI: 8.79–12.9), with an AAPC of −3.40 (95% CI: −4.00 to −2.80). Western Europe declined from 55.38 (95% UI: 51.75–59.03) to 19.10 (95% UI: 17.36–20.98), with an AAPC of −3.40 (95% CI: −3.80 to −3.00), and Central Asia dropped from 59.58 (95% UI: 55.42–64) to 22.58 (95% UI: 19.73–25.61), with an AAPC of −3.20 (95% CI: −4.00 to −2.40). Corresponding ASMRs fell from 0.88 (95% UI: 0.79–0.99) to 0.27 (95% UI: 0.22–0.33), from 0.68 (95% UI: 0.59–0.76) to 0.23 (95% UI: 0.20–0.27), from 1.34 (95% UI: 1.21–1.47) to 0.47 (95% UI: 0.38–0.56), from 1.94 (95% UI: 1.81–2.07) to 0.72 (95% UI: 0.64–0.80), and from 1.99 (95% UI: 1.84–2.14) to 0.83 (95% UI: 0.73–0.94). The AAPCs were −3.70 (95% CI: −4.50 to −3.00), −3.50 (95% CI: −3.90 to −3.00), −3.30 (95% CI: −4.00 to −2.70), −3.20 (95% CI: −3.50 to −2.80), and −2.80 (95% CI: −3.60 to −2.00), respectively (Figure 3, Supplementary Figure S2).

At the national and regional levels, the most significant declines in ASDRs and ASMRs were observed in the Republic of Korea, Singapore, Kuwait, and Colombia. For ASDRs, the average annual percentage change (AAPC) was −5.20 (95% CI: −5.50 to −4.90) in the Republic of Korea, −5.00 (95% CI: −6.80 to −3.10) in Singapore, −4.80 (95% CI: −8.50 to −1.00) in Kuwait, and −4.50 (95% CI: −5.60 to −3.40) in Colombia. Correspondingly, the AAPC for ASMRs was −4.80 (95% CI: −5.00 to −4.60) in the Republic of Korea, −4.80 (95% CI: −7.10 to −2.40) in Singapore, −4.40 (95% CI: −8.00 to −0.60) in Kuwait, and −4.20 (95% CI: −5.40 to −3.10) in Colombia. In contrast, Lesotho, Chad, Guinea, and Guinea-Bissau represent a few exceptions where both ASDRs and ASMRs showed an increasing trend in average annual percentage change (AAPC). For ASDRs, the AAPC was 1.80 (95% CI: 1.40–2.20) in Lesotho, 1.20 (95% CI: 1.00–1.40) in Chad, 1.20 (95% CI: 1.10–1.30) in Guinea, and 1.00 (95% CI: 0.90–1.20) in Guinea-Bissau. Similarly, the AAPC for ASMRs was 1.60 (95% CI: 1.20–2.00) in Lesotho, 1.30 (95% CI: 1.10–1.40) in Chad, 1.20 (95% CI: 1.10–1.40) in Guinea, and 1.00 (95% CI: 0.80–1.20) in Guinea-Bissau (Supplementary Figure S3).

### Changes in age-standardized disease burden rates in relation to socio-economic indices

**Figure 4:**
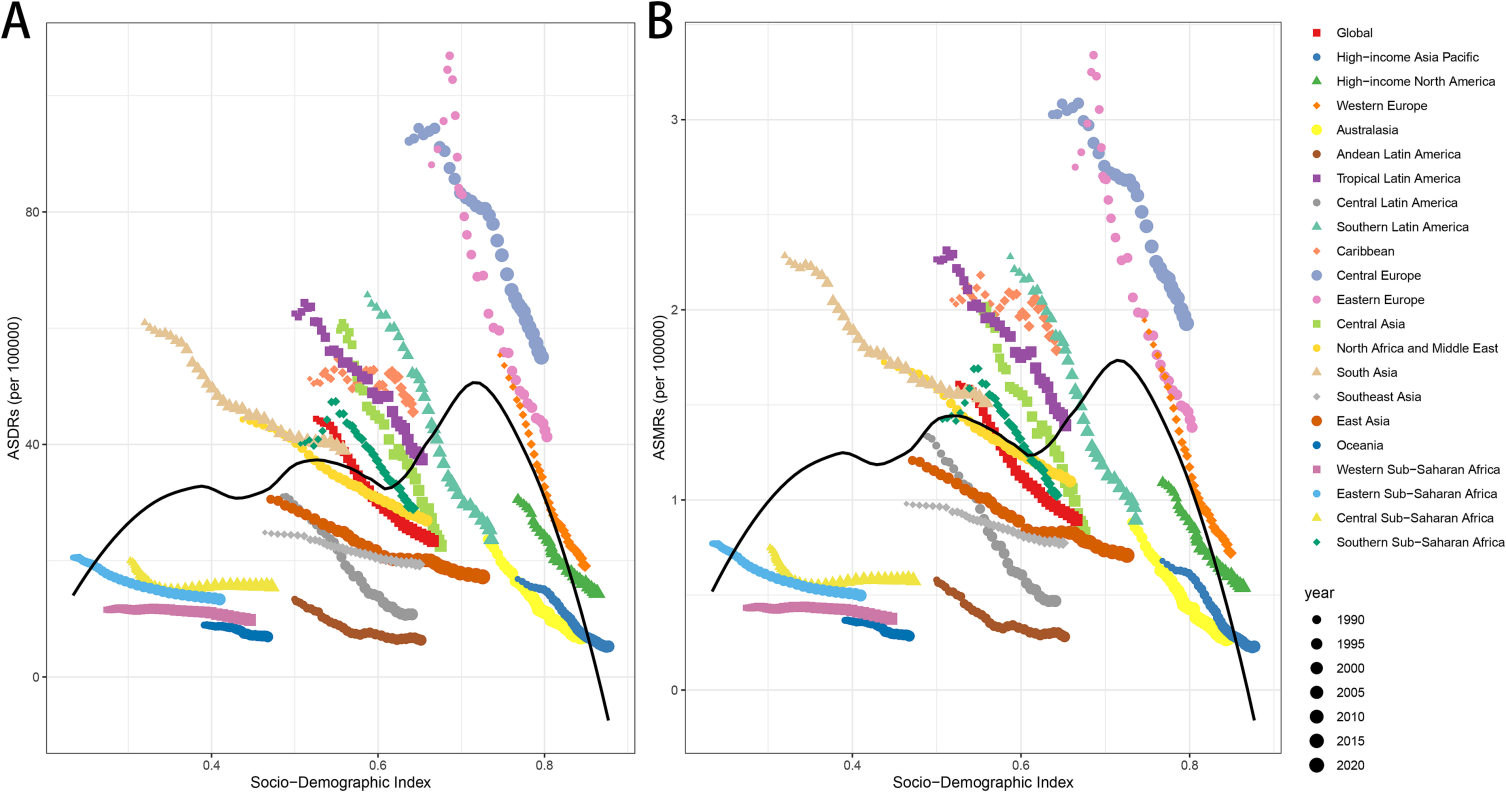
ASDRs and ASMRs of LCAS by Socio-demographic Index (SDI) across regions, 1990–2021. **(A)** ASDRs in relation to SDI. **(B)** ASMRs in relation to SDI. SDI is a composite index reflecting income, educational attainment, and total fertility rate.

The analysis of the correlation between the Socio-Demographic Index (SDI) and both ASDRs and ASMRs demonstrated a significant negative association globally and across most regions, reinforcing the relationship between higher SDI and reduced disease burdens. At the global level, the correlation coefficient for both ASDRs and ASMRs was −1 with a *p*-value of 0, highlighting a strong and consistent inverse relationship (Figures 4A,B). Regional analyses further validated this pattern, revealing pronounced negative correlations across various regions. Notably, the North Africa and Middle East region demonstrated a correlation of −1 (*p* = 0) for both ASMR and ASDR, highlighting a consistent and perfect inverse relationship. Similarly, strong associations were evident in High-income Asia Pacific, Western Europe, Southeast Asia, and Eastern Sub-Saharan Africa, with ASMR correlations of −0.9996 (*p* = 1.37E−48) and ASDR correlations of −0.9996 (*p* = 1.37E−48) for all these regions. Additionally, High-income North America, Australasia, Southern Latin America, and East Asia displayed substantial correlations, with ASMR values of −0.9993 (*p* = 4.48E−44) and ASDR values of −0.9993 (*p* = 4.48E−44). In contrast, less pronounced associations were observed in regions such as Western Sub-Saharan Africa (ASMR *r* = −0.73, *p* = 2.59E−06; ASDR *r* = −0.82, *p* = 1.33E−08), Southern Sub-Saharan Africa (ASMR *r* = −0.80, *p* = 5.45E−08; ASDR *r* = −0.79, *p* = 7.26E−08), Central Sub-Saharan Africa (ASMR *r* = −0.59, *p* = 0.0004; ASDR *r* = −0.62, *p* = 0.0002), and the Caribbean (ASMR *r* = −0.72, *p* = 3.70E−06; ASDR *r* = −0.49, *p* = 0.0047) (Figures 4A,B). These differences highlight the varying degrees of correlation across regions with distinct socio-demographic and healthcare profiles.

### Decomposition analysis of the driving factors behind DALYs and mortality burden

Globally, the rise in the number of smoking-attributable laryngeal cancer deaths and DALYs is largely driven by two key factors: aging (DALYs: +111.71%, Deaths: +81.46%) and population growth (DALYs: +626.61%, Deaths: +358.65%). In contrast, epidemiological changes have a significant role in mitigating the disease burden (DALYs: −638.32%, Deaths: −340.11%) (Supplementary Tables S3, S4). From a regional perspective, regions such as High-income North America, High-income Asia Pacific, Australasia, Central Europe, Western Europe, Eastern Europe, Southern Latin America, and Central Asia are the few areas where both DALYs and deaths have simultaneously decreased. In these regions, both aging and population growth contribute negatively to disease burden, meaning that population aging and growth together drive an increase in DALYs and death counts. Notably, High-income Asia Pacific is the only region globally where the rise in disease burden is mainly driven by population aging (DALYs: −96.52%, Deaths: −410.93%), significantly outweighing the influence of population growth (DALYs: −93.71%, Deaths: −260.97%). In contrast, other regions show a larger contribution of population growth to disease burden. This is especially evident in African regions such as Eastern Sub-Saharan Africa, Western Sub-Saharan Africa, and Central Sub-Saharan Africa, where the contribution of population growth to DALYs is 236.07, 142.57, and 154.44%, respectively, while the contribution of population aging to DALYs is negative: −20.78, −13.77%, and −12.65%. This suggests that the lower average life expectancy in these regions has a negative impact on disease burden. In Latin American regions (Tropical Latin America, Andean Latin America, Central Latin America) and Southeast Asia, the influence of population growth on disease burden is also more pronounced compared to other regions. Specifically, the contribution of population growth to DALYs is 179.07, 317.68, and 2095.62%, respectively, while for Deaths, the contribution is 154.1, 267.39, and 998.29%. In Southeast Asia, the contribution of population growth to DALYs is 114.12%, and to Deaths, it is 109.71% (Supplementary Tables S3, S4).

### Forecasting the global disease burden of LCAS in the next 15 years

**Figure 5:**
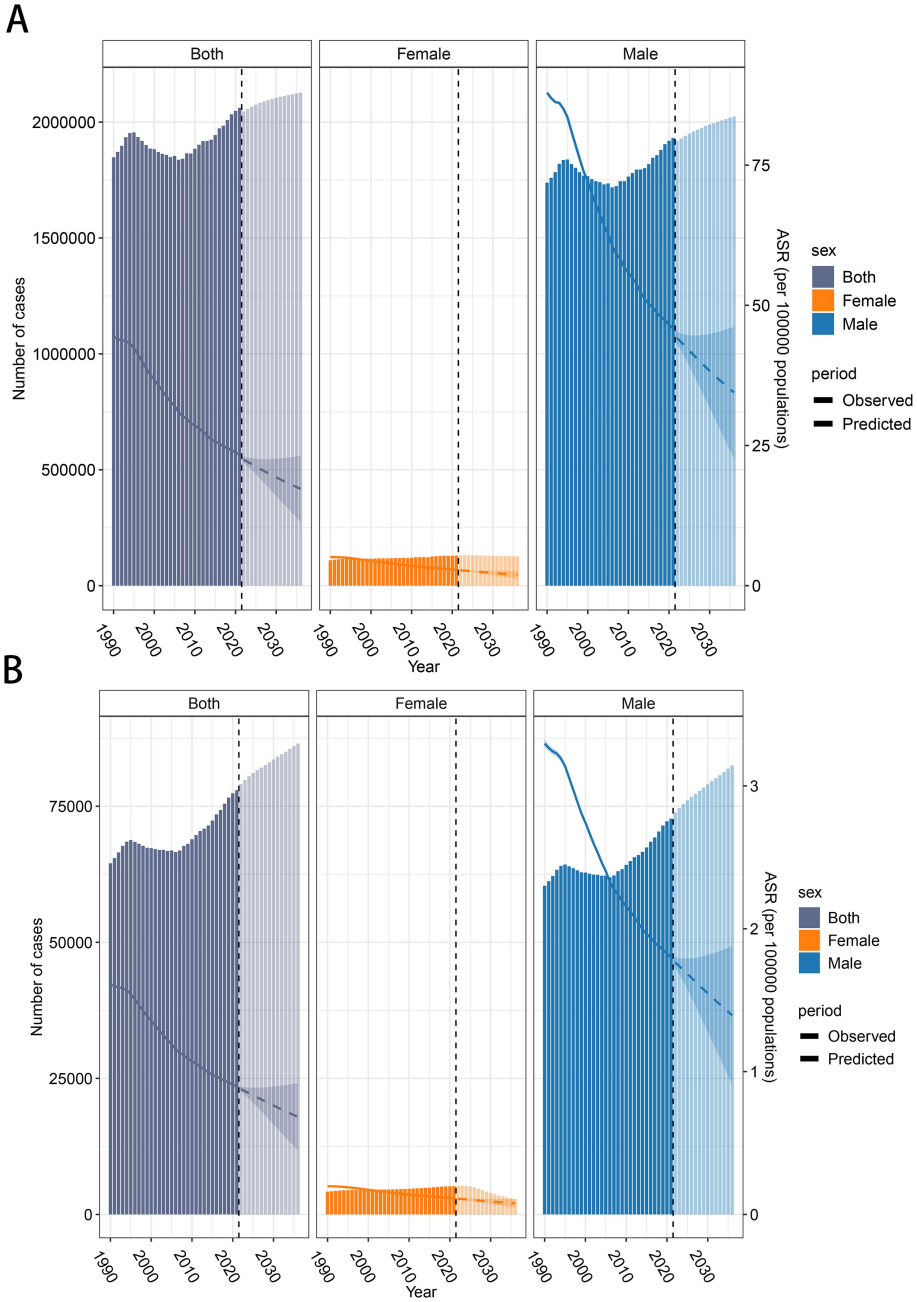
Global trends in the number of cases and age-standardized rates (ASR) of LCAS from 1990 to 2036. **(A)** DALY and ASDR trend of LCAS. **(B)** Deaths and ASMR trend of LCAS. The bar charts represent the number of cases, with 1990–2021 reflecting observed data and 2022–2036 showing predicted values. The line graphs illustrate the ASDR and ASMR trends, with solid lines representing observed ASR (1990–2021) and dashed lines representing predicted ASR (2022–2036).

Based on the BAPC prediction results, it is projected that the number of *DALYs* will rise from 2,061,113 in 2021 to 2,126,663 by 2036. Of this total, the number of DALYs for females is expected to decrease from 130,142 to 126,513, while for males, it is projected to rise from 1,930,971 to 2,024,011. The ASDR is expected to decrease, dropping from 23.38 in 2021 to 17.27 in 2036. Specifically, the ASDR for males will decline from 45.86 to 34.45, while females will experience a reduction from 2.83 to 1.94(Figure 5A). In terms of mortality, the number of deaths is anticipated to rise from 77,951 in 2021 to 86,524 in 2036. The male mortality number will increase from 72,740 to 82,497 over the same period. However, for females, the death toll is expected to decrease substantially, from 5,211 in 2021 to 2,818 by 2036. The ASMR is projected to decline from 0.895 in 2021 to 0.683 in 2036. Specifically, for males, it will decrease from 1.80 to 1.394, and for females, from 0.11 to 0.077(Figure 5B).

## Discussion

Leveraging the Global Burden of Disease (GBD) 2021 database, this study systematically analyzed the burden of smoking-related laryngeal cancer (LCAS) from 1990 to 2021, employing temporal trend analysis, decomposition methods, and Bayesian Age-Period-Cohort (BAPC) projections. Our findings reveal a slight increase in global LCAS burden, with DALYs reaching 2,061,113 (95% UI: 1,838,588–2,281,339) in 2021, predominantly driven by males (e.g., Central Europe male ASMR: 4.0 vs. female 0.3 per 100,000). However, age-standardized rates (ASDR: 23.38; ASMR: 0.89 per 100,000) declined globally and regionally reflecting strengthened tobacco control measures. The burden of LCAS varies markedly by age, region, and socioeconomic factors. ASDR peaked among individuals aged 65–74 years, while ASMR were highest at ages 70–84, reflecting prolonged exposure to risk factors like smoking. South Asia, East Asia, and Western Europe exhibited the heaviest burdens, with India (444,574 DALYs, 95% UI: 360,630–531,702), China, and Pakistan bearing the highest national loads, likely due to persistent tobacco use. A notable negative correlation between ASDR/ASMR and the SDI suggests that higher socioeconomic development reduces LCAS burden, consistent with improved access to prevention. However, regional differences in burden drivers, such as smoking prevalence and healthcare access, highlight the need for tailored interventions, particularly in South Asia where burdens remain elevated. Projections indicate a modest rise in global DALYs (from 2,061,113 in 2021) and male deaths by 2036, driven by aging populations, while female deaths may decline, underscoring gender-specific trends that warrant targeted tobacco control strategies.

The tobacco epidemic represents a preventable health risk. It has been reported that tobacco—induced deaths account for approximately 700,000 fatalities annually in Europe (20). Despite the implementation of an array of measures over the past two decades in the European region, among which is the enforcement of the Framework Convention on Tobacco Control, which, in turn, has contributed to a discernible decline in the overall smoking rate across the continent, Europe still persists as the region boasting the highest global smoking rate, with a figure standing at 29% (21). This can evidently explain why, in our study, the ASDRs and ASMRs in Central and Eastern Europe have significantly decreased over the past 30 years, yet still remain significantly higher compared to other regions. Specifically, within the context of European smoking prevalence, the rates in selected countries are as follows: Serbia stands at 39.8%, Montenegro at 32.8%, Bulgaria at 39.0%, Georgia at 31.7%, Hungary at 31.8%, Greece at 36.6%, and Sweden at 7.2% (22). Notably, Sweden, which holds the lowest ASDR and ASMR among Western European countries, which aligns with the results of our study. Across various regions globally, the ASDRs and ASMRs of LCAS in men exceed those in women by more than 10 times. This finding is consistent with the results of a retrospective study in South Korea involving 9,598,085 participants, which indicated that the risk of laryngeal cancer in men is 10.981 times that in women (23). Intriguingly, in most countries, the male smoking rate is less than twice that of females (24). Moreover, the overall prognosis for laryngeal cancer is currently comparable between men and women (25). This phenomenon may potentially be attributed to the crucial role that sex hormones might play in the carcinogenic process and cancer susceptibility under the same risk factors. Specifically, androgens are significantly associated with a higher cancer prevalence in men, while estrogens appear to have a protective effect on the disease burden of laryngeal cancer in women. This is also in line with the result that, even after adjusting for smoking factors, the incidence of laryngeal cancer in men remains higher than that in women (23). Furthermore, gender—related differences in humoral and cell—mediated immune responses to viral infections may partially account for the disparities in the disease burden (26).

Analysis of the LCAS burden across age groups reveals that high ASDRs and ASMRs are predominantly concentrated among individuals aged 60 and above. It is well—known that smoking has a cumulative—dose characteristic and is significantly and negatively correlated with the prognosis of diseases, including lung cancer (27). We postulate that with the improvement of economic levels and the increase in life expectancy, the cumulative dose of smoking among smokers gradually rises. This leads to an elevated risk of smoking—induced opportunistic carcinogenesis, which in turn naturally results in an increase in ASDRs and ASMRs. And this precisely explains the phenomenon in our results, where population aging has been the primary driver of the DALYs and the number of deaths in high—income Asia—Pacific regions (Japan, South Korea, Singapore, Brunei) over the past 30 years. Another aspect is that there is a strong association between the process of aging itself and cancer susceptibility. The main underlying mechanisms may be as follows: Telomere length decreases with advancing age, and short telomeres are associated with genomic instability (28). This instability promotes the occurrence of tumors. Secondly, aging leads to a decline in the ability of immune cells, including T—cells, to recognize novel antigens, alterations in immune function, and reduced T—cell activation. These changes result in a weakened anti—tumor response, thereby facilitating carcinogenesis (29). Additionally, the physical capacity and frailty status in the older adult, which affect this process, are also significant factors contributing to the increase in ASDRs and ASMRs (30).However, the phenomenon observed in our study, where ASDRs and ASMRs decrease among individuals aged over 90, may be related to the “antagonistic” effect between telomere attrition, stem cell exhaustion, and tumorigenesis. Telomere wear—and—tear and stem cell depletion might create a situation where they counteract the development of tumors (31). Certainly, in addition to the mentioned regions, including Australasia, Central Asia, Central Europe, Eastern Europe, High—income Asia Pacific, High—income North America, and Western Europe, all are experiencing population aging to varying degrees. This demographic shift exerts a negative feedback effect on the reduction of DALYs and the number of deaths in these regions. Therefore, more proactive measures should be implemented to address the issue of population aging.

The correlation analysis between social indices (SDI) and ASDRs as well as ASMRs indicates that as the SDI increases, ASDRs and ASMRs decline significantly in our study. Research has confirmed that individuals who are homeless, have low incomes, and a lower level of education have a higher smoking prevalence. Conversely, the smoking prevalence among the population in affluent areas is relatively lower (32). A survey of 16,925 participants in Nepal has found that, regardless of gender, the smoking prevalence in poor families is two to three times that in the wealthiest families (33). Over the past 30 years, the medical standards brought about by socioeconomic development have not significantly improved the overall survival of laryngeal cancer (34). However, there has been a decline in the ASDRs and ASMRs of LCAS. This can likely be attributed to the fact that with economic development, the enhancement of public health awareness, and the implementation of tobacco control frameworks in various regions, the smoking rate has been controlled to a certain extent. This control plays a facilitating role in reducing the disease burden caused by tobacco (21). Of course, socioeconomic development also brings about increased accessibility to medical facilities. This promotes the screening of LCAS, which can effectively reduce LCAS burden. The economic burden inflicted by smoking persists at a high level globally, with Europe being the region most significantly affected, where the costs arising from smoking—induced diseases amount to 2.5% of the region’s annual GDP; among European sub—regions, the situation in Eastern Europe is particularly striking, as the costs there account for a staggering 3.6% of the GDP, in contrast to merely 2.0% in the rest of Europe. Similarly, in Canada and the United States, which together bear a combined cost equivalent to 3.0% of their GDP due to smoking—related illnesses (35). This further emphasizes the importance of tobacco control in reducing the global burden of LCAS.

In Singapore and South Korea, the decline in ASDR (Age—Standardized Death Rate) and ASMR (Age—Standardized Mortality Rate) has been most remarkable, which is in line with previous reports (3). This is attributed to the governments’ efforts in restricting cigarette promotion while gradually expanding smoking restrictions. Additionally, the advanced medical standards and the implementation of early cancer screening programs for tumors have effectively reduced ASDRs and ASMRs (36, 37). However, in contrast to the global trend, the AAPC of ASDRs and ASMRs in Lesotho, Chad, Guinea, Guinea—Bissau, Ghana, Sao Tome and Principe, Burkina Faso, and Cuba are showing a tendency to increase. These regions mainly include African countries and Cuba, a large—scale smoking country. A study in Cuba with 146,556 participants found that the smoking rate among men is as high as 52%, while that among women is 29% (38). In the African region, the main issue is that the policies of the Framework Convention on Tobacco Control regarding monitoring, advertising, and smoking bans have not been effectively implemented in most African countries. Moreover, from 1980 to 2016, tobacco consumption in the African region increased by more than 50% (39). From another perspective, it also underlines the pressing urgency for Cuba and African regions to boost investment in healthcare and intensify the screening efforts for laryngeal cancer.

The limitations of our study should also be recognized. First, the estimation of the LCAS burden highly depends on the availability and quality of GBD 2021 data. In some countries, particularly low—and middle—income countries, due to the imperfect cancer screening systems, it may be impossible to obtain original and accurate data, which is likely to affect the data estimation results of GBD researchers. Second, the information on some key influencing factors in the current dataset may not be detailed enough. For example, although we know that smoking is related to LCAS, there is a lack of comprehensive statistics on details such as the specific types of cigarettes smoked (such as flue—cured tobacco, sun—cured tobacco, etc.), smoking methods (such as filter use), and the distribution of smokers’ smoking years. This limits our ability to analyze the precise impact of smoking on the LCAS burden.

## Conclusion

From 1990 to 2021, various regions around the world have made numerous efforts in reducing the burden of LCAS, achieving remarkable results. The ASDRs and ASMRs in all regions have shown a significant downward trend. Among them, Europe, high—income Asia—Pacific regions, and North America have achieved particularly outstanding results in reducing DALYs and death burdens. However, disparities persist conspicuously among regions of varying income levels. Low—income African regions, in particular, encounter more acute challenges in this context. Given the crucial role of tobacco use in the onset of LCAS, future work should focus on enhancing public awareness of the hazards of tobacco. Especially in low—income regions, it is necessary to strengthen tobacco control measures and vigorously enhance early screening for laryngeal cancer. In addition, increasing investment in medical infrastructure in low—income regions and improving the accessibility of medical services are of vital importance for narrowing regional gaps and achieving global health equity.

## Data Availability

Publicly available datasets were analyzed in this study. This data can be found here: https://www.healthdata.org/research-analysis/gbd.

## Footnotes

^1^ http://ghdx.healthdata.org/
