# Supplementary material for: Global, regional, and national burden of laryngeal cancer attributable to smoking, 1990–2021, and projections to 2036: a systematic analysis of the Global Burden of Disease study 2021
Source: Front Public Health. 2025 May 9;13:1583045. doi: 10.3389/fpubh.2025.1583045 (PMC12098609; doi:10.3389/fpubh.2025.1583045)
Supplement: Supplementary file 1 [file Data_Sheet_1.docx]

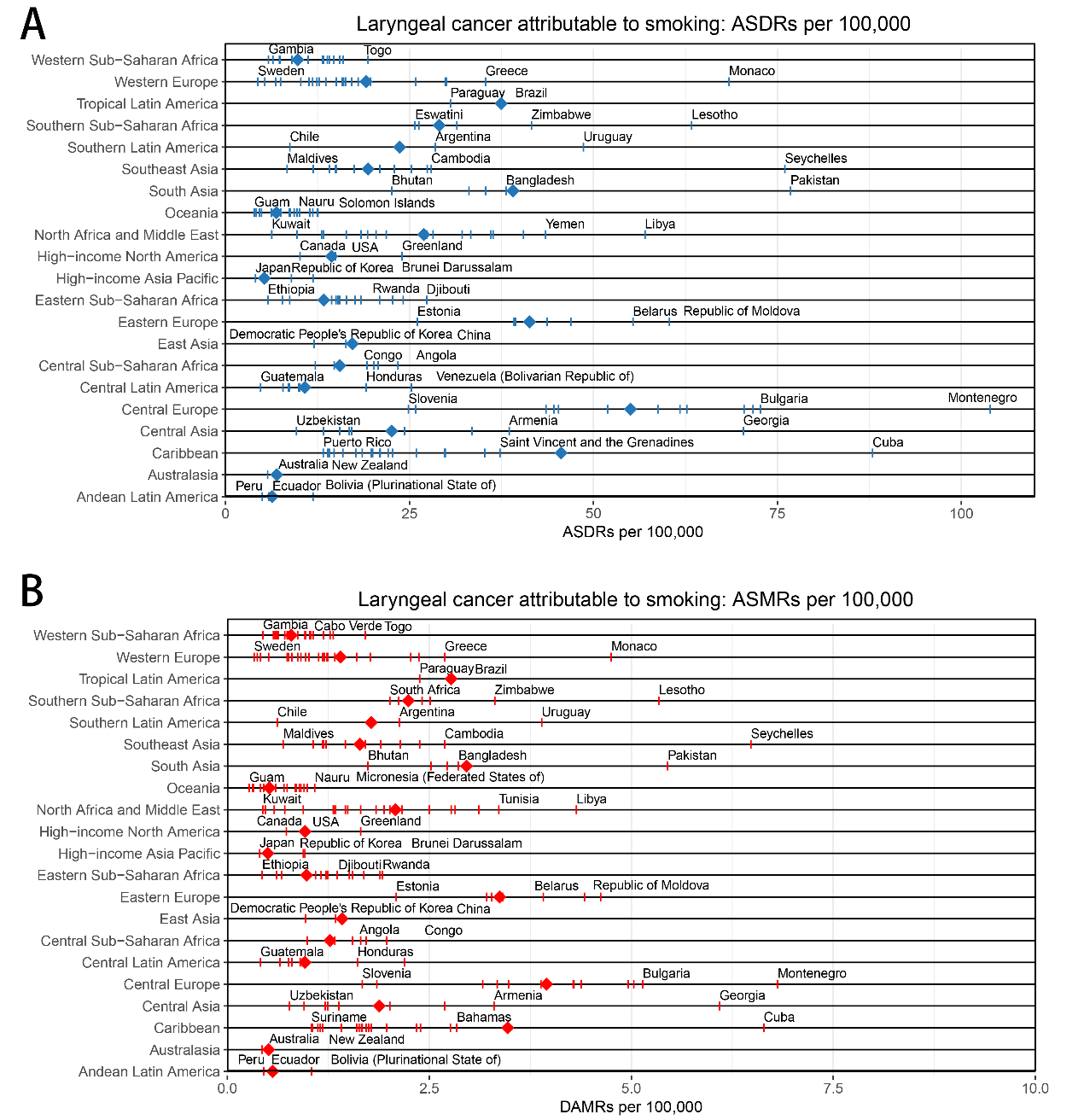


**Figure S1. Smoking-Attributed Laryngeal Cancer ASMR and ASDR per 100,000 by World Regions, Countries, and Territories in 2021.** Diamond Shapes Represent Regional Levels, Vertical Lines Represent Countries or Territories.


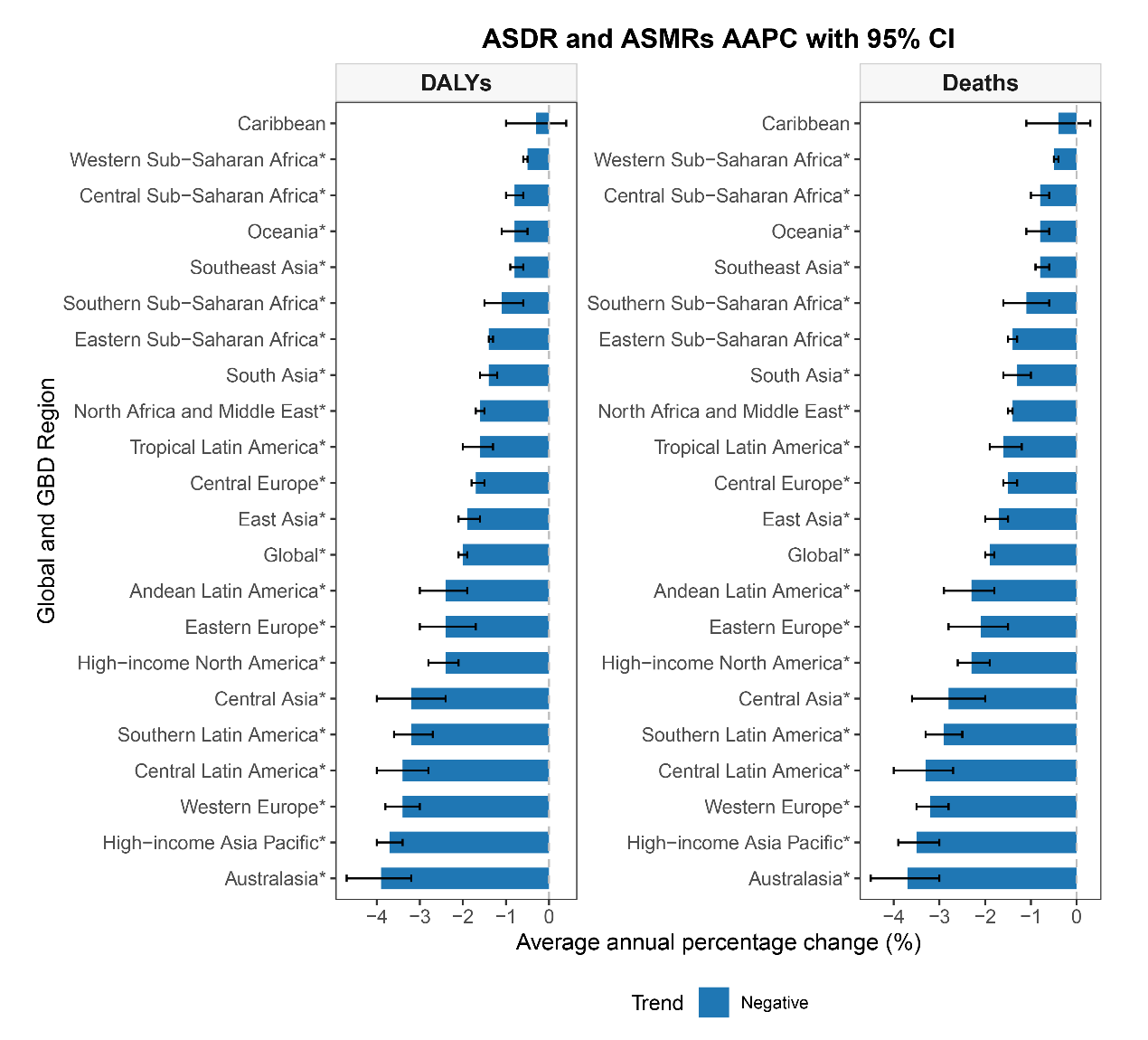


**Figure S2. Annual Percentage Change (AAPC) of LCAS ASDRs and ASMRs in Global and Regional Contexts, 1990–2021.** The asterisk (*) after the place name indicates a significant statistical difference.


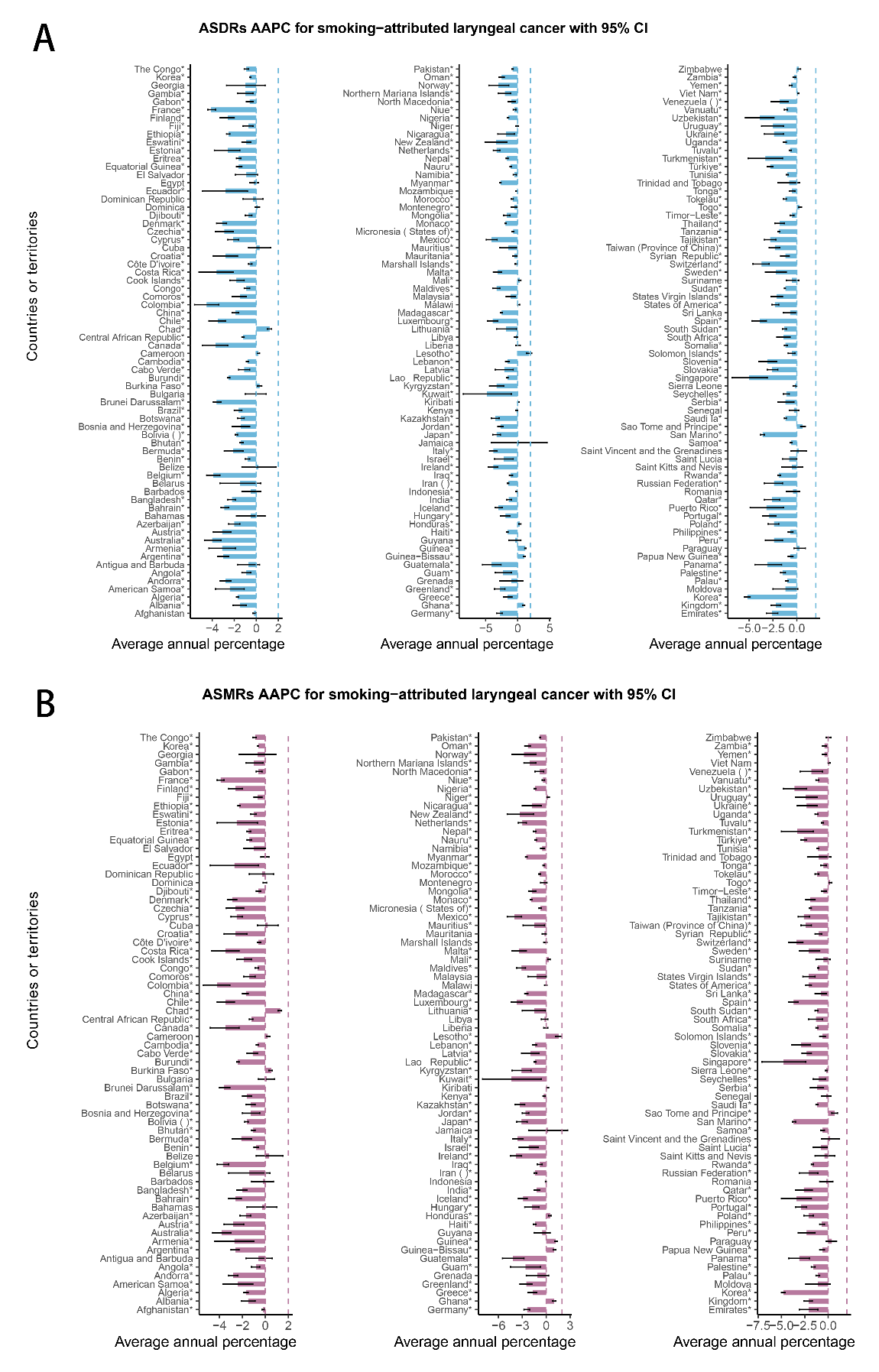


**Figure S3. Annual Percentage Change (AAPC) of LCAS ASDRs and ASMRs in 204 countries and territories 1990–2021.** The asterisk (*) after the place name indicates a significant statistical difference.

**Table S1. Mortality and DALYs of LCAS in 2021: 204 countries or territories**

| Location_name | Year | DALYs (UI) | ASDRs (UI) | Motality (UI) | ASMRs (UI) |
| --- | --- | --- | --- | --- | --- |
| Afghanistan | 2021 | 3688 (2008,5669) | 32.16 (18.24,47.51) | 120 (68,178) | 1.26 (0.74,1.8) |
| Albania | 2021 | 1855 (1272,2683) | 43.54 (30.27,61.83) | 73 (49,105) | 1.66 (1.14,2.38) |
| Algeria | 2021 | 7525 (5249,10292) | 20.45 (14.49,27.83) | 287 (203,390) | 0.89 (0.64,1.19) |
| American Samoa | 2021 | 4 (3,6) | 8.67 (6.57,11.21) | 0 (0,0) | 0.37 (0.28,0.49) |
| Andorra | 2021 | 21 (13,30) | 13.62 (8.23,19.8) | 1 (0,1) | 0.49 (0.3,0.71) |
| Angola | 2021 | 3172 (2300,4188) | 23.43 (17.31,30.69) | 105 (78,138) | 0.89 (0.66,1.17) |
| Antigua and Barbuda | 2021 | 26 (21,30) | 22.67 (18.67,26.64) | 1 (1,1) | 0.93 (0.77,1.1) |
| Argentina | 2021 | 15485 (13458,17704) | 28.52 (24.86,32.58) | 602 (515,690) | 1.08 (0.93,1.24) |
| Armenia | 2021 | 1682 (1492,1896) | 38.55 (34.22,43.49) | 63 (56,71) | 1.42 (1.27,1.6) |
| Australia | 2021 | 2984 (2427,3546) | 7.2 (5.86,8.53) | 126 (101,153) | 0.28 (0.23,0.34) |
| Austria | 2021 | 2587 (2221,2951) | 16.03 (13.8,18.3) | 106 (90,121) | 0.61 (0.52,0.69) |
| Azerbaijan | 2021 | 4021 (2886,5957) | 33.48 (24.16,49) | 134 (97,196) | 1.2 (0.87,1.74) |
| Bahamas | 2021 | 157 (117,197) | 35.24 (26.32,44.09) | 6 (4,7) | 1.32 (0.99,1.66) |
| Bahrain | 2021 | 216 (156,320) | 21.84 (15.8,30.99) | 7 (5,11) | 0.99 (0.73,1.38) |
| Bangladesh | 2021 | 54580 (38472,75573) | 38.12 (27.04,52.12) | 2062 (1480,2799) | 1.52 (1.09,2.05) |
| Barbados | 2021 | 92 (65,122) | 17.7 (12.66,23.56) | 4 (3,5) | 0.76 (0.55,1) |
| Belarus | 2021 | 8606 (6447,11100) | 55.37 (41.51,70.7) | 291 (221,372) | 1.81 (1.38,2.3) |
| Belgium | 2021 | 3922 (3453,4444) | 19.38 (17.05,21.93) | 159 (139,182) | 0.71 (0.63,0.81) |
| Belize | 2021 | 70 (58,85) | 22.09 (18.19,26.9) | 3 (2,3) | 0.88 (0.72,1.07) |
| Benin | 2021 | 487 (338,652) | 9.02 (6.26,11.96) | 18 (12,23) | 0.35 (0.24,0.46) |
| Bermuda | 2021 | 27 (21,34) | 20.94 (16.4,26.29) | 1 (1,1) | 0.82 (0.65,1.04) |
| Bhutan | 2021 | 138 (87,204) | 22.59 (14.22,33.06) | 6 (4,8) | 0.95 (0.62,1.4) |
| Bolivia (Plurinational State of) | 2021 | 1108 (720,1673) | 11.88 (7.75,17.66) | 45 (29,66) | 0.52 (0.34,0.75) |
| Bosnia and Herzegovina | 2021 | 3518 (2513,4619) | 58.76 (41.6,77.1) | 135 (96,177) | 2.17 (1.55,2.83) |
| Botswana | 2021 | 476 (318,758) | 29.1 (19.71,45.37) | 16 (11,25) | 1.1 (0.75,1.64) |
| Brazil | 2021 | 97517 (85520,109503) | 37.61 (32.96,42.26) | 3564 (3124,4020) | 1.4 (1.22,1.58) |
| Brunei Darussalam | 2021 | 43 (32,57) | 11.88 (9.12,15.71) | 2 (1,2) | 0.52 (0.4,0.68) |
| Bulgaria | 2021 | 8967 (6950,11245) | 72.69 (56.24,91.22) | 324 (251,409) | 2.45 (1.9,3.09) |
| Burkina Faso | 2021 | 959 (602,1356) | 9.54 (5.93,13.36) | 33 (20,46) | 0.35 (0.22,0.49) |
| Burundi | 2021 | 802 (516,1167) | 14.41 (9.33,20.72) | 27 (17,38) | 0.53 (0.35,0.76) |
| Cabo Verde | 2021 | 67 (44,93) | 14.08 (9.18,19.27) | 2 (2,3) | 0.55 (0.36,0.76) |
| Cambodia | 2021 | 3690 (2420,5804) | 27.96 (18.5,43.4) | 138 (91,212) | 1.15 (0.76,1.73) |
| Cameroon | 2021 | 1968 (1200,2934) | 13.83 (8.47,20.47) | 65 (40,97) | 0.5 (0.31,0.75) |
| Canada | 2021 | 6938 (6009,8045) | 10.12 (8.74,11.74) | 298 (253,347) | 0.4 (0.34,0.47) |
| Central African Republic | 2021 | 541 (302,864) | 20.17 (11.89,31.03) | 17 (10,27) | 0.73 (0.46,1.09) |
| Chad | 2021 | 901 (588,1274) | 14.72 (9.57,20.76) | 32 (21,46) | 0.58 (0.38,0.83) |
| Chile | 2021 | 2207 (1866,2608) | 8.73 (7.41,10.28) | 81 (66,99) | 0.32 (0.26,0.38) |
| China | 2021 | 381556 (284584,497331) | 17.34 (12.97,22.59) | 15274 (11616,19867) | 0.72 (0.55,0.93) |
| Colombia | 2021 | 4716 (3544,6134) | 8.52 (6.41,11.1) | 207 (156,270) | 0.38 (0.28,0.49) |
| Comoros | 2021 | 86 (59,126) | 16.45 (11.24,23.53) | 3 (2,4) | 0.65 (0.46,0.91) |
| Congo | 2021 | 622 (441,867) | 20.73 (14.85,28.42) | 21 (15,29) | 0.82 (0.59,1.12) |
| Cook Islands | 2021 | 1 (1,2) | 4.89 (3.55,6.84) | 0 (0,0) | 0.21 (0.15,0.29) |
| Costa Rica | 2021 | 550 (444,661) | 9.93 (8.02,11.92) | 24 (19,28) | 0.43 (0.35,0.52) |
| Côte d'Ivoire | 2021 | 2008 (1314,3102) | 15.47 (10.25,23.38) | 66 (44,99) | 0.57 (0.39,0.85) |
| Croatia | 2021 | 3587 (2964,4233) | 45.25 (37.41,53.5) | 143 (119,168) | 1.67 (1.38,1.95) |
| Cuba | 2021 | 17080 (13704,20882) | 87.94 (70.5,106.76) | 674 (545,822) | 3.41 (2.76,4.14) |
| Cyprus | 2021 | 303 (232,394) | 15.05 (11.61,19.63) | 12 (9,16) | 0.58 (0.45,0.75) |
| Czechia | 2021 | 4882 (3824,6125) | 25.85 (20.2,32.4) | 188 (148,235) | 0.93 (0.73,1.16) |
| Democratic People's Republic of Korea | 2021 | 5720 (3867,7902) | 16.36 (11.17,22.58) | 199 (142,269) | 0.59 (0.42,0.79) |
| Democratic Republic of the Congo | 2021 | 5202 (3400,7521) | 12.2 (7.93,17.51) | 171 (111,246) | 0.45 (0.29,0.63) |
| Denmark | 2021 | 1848 (1636,2092) | 17.15 (15.13,19.37) | 80 (70,91) | 0.68 (0.6,0.77) |
| Djibouti | 2021 | 205 (132,304) | 27.33 (18.01,39.13) | 7 (4,10) | 1.05 (0.7,1.48) |
| Dominica | 2021 | 26 (19,35) | 29.86 (21.28,39.97) | 1 (1,1) | 1.15 (0.82,1.54) |
| Dominican Republic | 2021 | 2621 (1793,3601) | 25.91 (17.78,35.59) | 104 (73,142) | 1.05 (0.74,1.43) |
| Ecuador | 2021 | 967 (693,1287) | 5.9 (4.23,7.84) | 42 (31,55) | 0.26 (0.19,0.34) |
| Egypt | 2021 | 12761 (9451,17475) | 18.4 (13.87,24.86) | 446 (334,608) | 0.75 (0.58,1) |
| El Salvador | 2021 | 469 (335,635) | 7.8 (5.59,10.56) | 18 (13,25) | 0.3 (0.21,0.41) |
| Equatorial Guinea | 2021 | 83 (47,131) | 14.76 (8.55,22.94) | 3 (2,4) | 0.57 (0.34,0.86) |
| Eritrea | 2021 | 534 (329,817) | 15 (9.38,22.69) | 16 (10,24) | 0.51 (0.32,0.77) |
| Estonia | 2021 | 587 (467,714) | 26.07 (20.85,31.85) | 22 (17,26) | 0.9 (0.71,1.09) |
| Eswatini | 2021 | 163 (92,242) | 25.75 (14.83,37.94) | 5 (3,8) | 0.92 (0.55,1.33) |
| Ethiopia | 2021 | 2667 (1694,3887) | 5.77 (3.7,8.39) | 95 (61,137) | 0.22 (0.14,0.32) |
| Fiji | 2021 | 76 (52,106) | 9.32 (6.61,12.79) | 3 (2,4) | 0.39 (0.28,0.52) |
| Finland | 2021 | 778 (658,915) | 7.47 (6.4,8.69) | 31 (26,37) | 0.26 (0.22,0.31) |
| France | 2021 | 29802 (25678,34674) | 25.87 (22.36,29.78) | 1160 (987,1359) | 0.91 (0.78,1.05) |
| Gabon | 2021 | 226 (152,317) | 19.22 (13.15,26.96) | 8 (5,11) | 0.73 (0.5,1.01) |
| Gambia | 2021 | 61 (43,83) | 5.82 (4.14,7.81) | 2 (1,3) | 0.21 (0.15,0.29) |
| Georgia | 2021 | 4024 (3443,4607) | 70.36 (60.16,80.55) | 151 (130,174) | 2.56 (2.2,2.94) |
| Germany | 2021 | 27318 (24194,30923) | 16.29 (14.42,18.34) | 1135 (990,1302) | 0.62 (0.54,0.7) |
| Ghana | 2021 | 1752 (1167,2430) | 9.94 (6.73,13.68) | 64 (43,88) | 0.41 (0.28,0.55) |
| Greece | 2021 | 7045 (6326,7759) | 35.33 (31.8,38.74) | 309 (276,342) | 1.35 (1.22,1.49) |
| Greenland | 2021 | 19 (15,25) | 23.97 (18.47,32.07) | 1 (1,1) | 0.91 (0.7,1.22) |
| Grenada | 2021 | 19 (15,22) | 14.75 (11.62,18.02) | 1 (1,1) | 0.55 (0.44,0.68) |
| Guam | 2021 | 8 (7,10) | 3.92 (3.21,4.82) | 0 (0,0) | 0.14 (0.11,0.17) |
| Guatemala | 2021 | 528 (414,666) | 4.76 (3.74,6) | 22 (17,28) | 0.21 (0.16,0.26) |
| Guinea | 2021 | 912 (637,1243) | 15.54 (10.99,20.97) | 33 (24,45) | 0.61 (0.44,0.81) |
| Guinea-Bissau | 2021 | 132 (82,194) | 15.96 (10.09,23.11) | 4 (3,6) | 0.58 (0.37,0.83) |
| Guyana | 2021 | 114 (78,160) | 15.93 (11.05,22.29) | 4 (3,5) | 0.57 (0.4,0.79) |
| Haiti | 2021 | 1677 (1026,2469) | 21.02 (12.8,30.63) | 58 (35,84) | 0.81 (0.48,1.2) |
| Honduras | 2021 | 1232 (867,1634) | 19.1 (13.41,25.48) | 50 (35,66) | 0.83 (0.57,1.1) |
| Hungary | 2021 | 10387 (8390,12626) | 62.69 (50.5,76.59) | 369 (299,447) | 2.1 (1.7,2.56) |
| Iceland | 2021 | 36 (30,43) | 6.83 (5.72,8.07) | 1 (1,2) | 0.25 (0.21,0.29) |
| India | 2021 | 444574 (360630,531702) | 35.35 (28.74,42.2) | 16321 (13391,19365) | 1.37 (1.12,1.62) |
| Indonesia | 2021 | 45479 (31206,61253) | 17.46 (11.82,23.44) | 1660 (1122,2214) | 0.72 (0.48,0.95) |
| Iran (Islamic Republic of) | 2021 | 22279 (18953,25806) | 27.01 (22.95,31.35) | 819 (693,958) | 1.08 (0.91,1.27) |
| Iraq | 2021 | 9206 (6161,12544) | 36.08 (24.48,49.09) | 332 (225,451) | 1.47 (1,1.99) |
| Ireland | 2021 | 892 (759,1036) | 11.84 (10.04,13.76) | 36 (30,42) | 0.45 (0.39,0.53) |
| Israel | 2021 | 1421 (1207,1670) | 12.42 (10.56,14.59) | 57 (48,68) | 0.47 (0.4,0.56) |
| Italy | 2021 | 25035 (22135,27963) | 19.72 (17.54,21.9) | 1128 (969,1285) | 0.79 (0.69,0.89) |
| Jamaica | 2021 | 614 (439,867) | 19.99 (14.28,28.23) | 25 (18,35) | 0.8 (0.57,1.13) |
| Japan | 2021 | 14189 (12573,15703) | 4.07 (3.65,4.46) | 765 (653,853) | 0.18 (0.16,0.2) |
| Jordan | 2021 | 1122 (794,1568) | 13.33 (9.41,18.57) | 38 (27,53) | 0.53 (0.38,0.74) |
| Kazakhstan | 2021 | 4922 (4188,5704) | 24.34 (20.69,28.22) | 163 (139,190) | 0.84 (0.71,0.97) |
| Kenya | 2021 | 3981 (2702,5451) | 15.42 (10.54,21) | 135 (92,183) | 0.58 (0.4,0.78) |
| Kiribati | 2021 | 3 (2,5) | 4.17 (2.88,5.86) | 0 (0,0) | 0.17 (0.12,0.23) |
| Kuwait | 2021 | 204 (158,260) | 6.27 (4.91,8.03) | 7 (6,9) | 0.27 (0.21,0.35) |
| Kyrgyzstan | 2021 | 929 (702,1206) | 16.81 (12.76,21.8) | 31 (23,40) | 0.6 (0.46,0.77) |
| Lao People's Democratic Republic | 2021 | 1019 (667,1541) | 20.94 (13.86,31.42) | 38 (25,56) | 0.86 (0.59,1.28) |
| Latvia | 2021 | 1291 (1059,1532) | 39.4 (32.28,46.97) | 47 (39,57) | 1.34 (1.1,1.6) |
| Lebanon | 2021 | 2123 (1649,2713) | 36.35 (28.12,46.63) | 90 (70,113) | 1.49 (1.16,1.87) |
| Lesotho | 2021 | 757 (500,1040) | 63.35 (42.25,86.25) | 25 (17,34) | 2.2 (1.49,2.96) |
| Liberia | 2021 | 232 (149,346) | 9.91 (6.45,14.77) | 8 (5,11) | 0.37 (0.24,0.55) |
| Libya | 2021 | 3411 (2352,4747) | 57.01 (39.7,78.45) | 115 (81,159) | 2.19 (1.55,2.97) |
| Lithuania | 2021 | 2294 (1791,2858) | 46.93 (36.64,58.13) | 85 (66,105) | 1.62 (1.27,2.01) |
| Luxembourg | 2021 | 182 (157,210) | 18.02 (15.56,20.8) | 7 (6,8) | 0.67 (0.58,0.78) |
| Madagascar | 2021 | 1159 (779,1663) | 8.72 (5.86,12.65) | 37 (25,54) | 0.33 (0.22,0.48) |
| Malawi | 2021 | 636 (450,922) | 7.72 (5.55,11.02) | 21 (16,30) | 0.29 (0.21,0.4) |
| Malaysia | 2021 | 6168 (4647,7665) | 20.98 (15.9,26.13) | 240 (183,296) | 0.87 (0.67,1.08) |
| Maldives | 2021 | 29 (21,39) | 8.37 (6.09,10.93) | 1 (1,2) | 0.39 (0.29,0.5) |
| Mali | 2021 | 951 (678,1371) | 10.09 (7.3,14.25) | 34 (25,47) | 0.41 (0.3,0.55) |
| Malta | 2021 | 125 (103,153) | 15.88 (13.17,19.48) | 5 (4,6) | 0.54 (0.45,0.66) |
| Marshall Islands | 2021 | 4 (3,6) | 11.44 (7.2,16.79) | 0 (0,0) | 0.48 (0.3,0.71) |
| Mauritania | 2021 | 256 (166,385) | 11.23 (7.27,16.63) | 9 (6,14) | 0.44 (0.29,0.64) |
| Mauritius | 2021 | 486 (431,537) | 25.27 (22.44,27.88) | 19 (17,21) | 0.99 (0.88,1.1) |
| Mexico | 2021 | 11071 (9036,13514) | 8.67 (7.07,10.57) | 478 (391,578) | 0.39 (0.32,0.47) |
| Micronesia (Federated States of) | 2021 | 10 (7,13) | 11.86 (8.46,16.26) | 0 (0,0) | 0.47 (0.33,0.64) |
| Monaco | 2021 | 54 (40,76) | 68.43 (50.1,97.31) | 2 (2,3) | 2.43 (1.78,3.41) |
| Mongolia | 2021 | 430 (290,616) | 15.49 (10.83,21.98) | 14 (10,19) | 0.55 (0.39,0.75) |
| Montenegro | 2021 | 1012 (756,1376) | 103.93 (77.66,141.07) | 37 (27,49) | 3.68 (2.76,4.97) |
| Morocco | 2021 | 10410 (7035,14023) | 28.2 (19.13,37.83) | 379 (258,502) | 1.08 (0.73,1.42) |
| Mozambique | 2021 | 2664 (1918,3687) | 20.92 (15.06,28.48) | 88 (63,119) | 0.77 (0.56,1.04) |
| Myanmar | 2021 | 7154 (4825,10287) | 14.1 (9.7,20.15) | 280 (196,400) | 0.6 (0.43,0.83) |
| Namibia | 2021 | 471 (332,643) | 31.44 (22.7,42.53) | 17 (12,22) | 1.21 (0.89,1.62) |
| Nauru | 2021 | 1 (0,1) | 12.55 (8.07,17.85) | 0 (0,0) | 0.5 (0.33,0.7) |
| Nepal | 2021 | 7983 (5683,11058) | 33.08 (23.77,45.85) | 310 (226,432) | 1.37 (1,1.91) |
| Netherlands | 2021 | 3765 (3279,4231) | 11.34 (9.87,12.78) | 164 (142,185) | 0.46 (0.4,0.51) |
| New Zealand | 2021 | 473 (389,558) | 5.71 (4.7,6.71) | 20 (16,25) | 0.24 (0.19,0.28) |
| Nicaragua | 2021 | 501 (375,673) | 10.08 (7.57,13.54) | 20 (15,27) | 0.43 (0.32,0.57) |
| Niger | 2021 | 539 (328,782) | 6.43 (4.03,9.24) | 20 (13,29) | 0.29 (0.19,0.42) |
| Nigeria | 2021 | 7637 (4877,10768) | 7.39 (4.81,10.43) | 260 (169,365) | 0.28 (0.18,0.39) |
| Niue | 2021 | 0 (0,0) | 7.53 (5.22,10.34) | 0 (0,0) | 0.31 (0.22,0.42) |
| North Macedonia | 2021 | 2473 (1801,3312) | 71.69 (52.2,96.19) | 89 (65,118) | 2.62 (1.92,3.45) |
| Northern Mariana Islands | 2021 | 5 (4,7) | 10.03 (7.9,12.2) | 0 (0,0) | 0.43 (0.34,0.53) |
| Norway | 2021 | 511 (434,585) | 5.34 (4.57,6.09) | 23 (19,27) | 0.22 (0.19,0.26) |
| Oman | 2021 | 148 (102,207) | 6.28 (4.44,8.53) | 5 (3,7) | 0.25 (0.18,0.34) |
| Pakistan | 2021 | 102554 (72353,139010) | 76.79 (54.06,104.02) | 3576 (2513,4837) | 3 (2.12,4.02) |
| Palau | 2021 | 2 (1,2) | 6.72 (4.66,9.17) | 0 (0,0) | 0.28 (0.19,0.39) |
| Palestine | 2021 | 443 (348,570) | 16.4 (13.01,20.76) | 16 (13,20) | 0.69 (0.55,0.86) |
| Panama | 2021 | 380 (282,488) | 8.63 (6.42,11.08) | 17 (13,22) | 0.38 (0.29,0.49) |
| Papua New Guinea | 2021 | 343 (217,534) | 6.23 (4.1,9.57) | 12 (8,18) | 0.26 (0.17,0.4) |
| Paraguay | 2021 | 1842 (1234,2610) | 30.56 (20.59,43.14) | 69 (47,97) | 1.19 (0.81,1.68) |
| Peru | 2021 | 1665 (1082,2432) | 4.99 (3.24,7.28) | 75 (49,107) | 0.23 (0.15,0.33) |
| Philippines | 2021 | 13238 (10325,16440) | 15.02 (11.8,18.54) | 480 (377,591) | 0.59 (0.47,0.72) |
| Poland | 2021 | 34727 (30409,39015) | 51.92 (45.64,58.27) | 1332 (1159,1504) | 1.89 (1.65,2.13) |
| Portugal | 2021 | 5916 (4990,6939) | 30.04 (25.55,34.99) | 230 (191,275) | 1.06 (0.89,1.25) |
| Puerto Rico | 2021 | 812 (617,1066) | 13.27 (9.94,17.44) | 35 (26,45) | 0.51 (0.38,0.66) |
| Qatar | 2021 | 227 (151,318) | 18.44 (12.1,26.62) | 7 (4,9) | 0.79 (0.52,1.14) |
| Republic of Korea | 2021 | 8559 (6237,11334) | 8.95 (6.55,11.85) | 391 (284,516) | 0.41 (0.3,0.54) |
| Republic of Moldova | 2021 | 3547 (3075,4043) | 60.33 (52.41,68.59) | 120 (104,136) | 1.99 (1.73,2.27) |
| Romania | 2021 | 22651 (18957,26634) | 70.46 (58.89,83.37) | 800 (667,936) | 2.35 (1.96,2.77) |
| Russian Federation | 2021 | 91580 (79782,101792) | 39.2 (34.19,43.58) | 3206 (2787,3561) | 1.33 (1.16,1.48) |
| Rwanda | 2021 | 1666 (1142,2478) | 24.15 (16.63,35.58) | 59 (41,87) | 0.95 (0.67,1.37) |
| Saint Kitts and Nevis | 2021 | 15 (12,20) | 19.79 (14.89,25.1) | 1 (0,1) | 0.78 (0.59,0.98) |
| Saint Lucia | 2021 | 74 (57,95) | 29.78 (22.94,38.26) | 3 (2,4) | 1.16 (0.89,1.5) |
| Saint Vincent and the Grenadines | 2021 | 55 (45,67) | 37.29 (30.19,45.35) | 2 (2,3) | 1.42 (1.13,1.74) |
| Samoa | 2021 | 7 (5,10) | 4.61 (3.45,6.41) | 0 (0,0) | 0.17 (0.13,0.23) |
| San Marino | 2021 | 10 (6,15) | 16.3 (9.41,24.57) | 0 (0,1) | 0.6 (0.36,0.89) |
| Sao Tome and Principe | 2021 | 9 (6,12) | 7.32 (5.22,9.83) | 0 (0,0) | 0.29 (0.21,0.39) |
| Saudi Arabia | 2021 | 2481 (1841,3257) | 9.7 (7.42,12.57) | 74 (56,97) | 0.36 (0.28,0.47) |
| Senegal | 2021 | 1127 (794,1533) | 13.33 (9.3,18.33) | 39 (27,54) | 0.49 (0.34,0.68) |
| Serbia | 2021 | 9275 (6311,13070) | 61.75 (41.79,87.77) | 349 (242,486) | 2.19 (1.5,3.07) |
| Seychelles | 2021 | 95 (75,117) | 76.01 (60.17,93.8) | 4 (3,4) | 3.03 (2.41,3.7) |
| Sierra Leone | 2021 | 533 (365,763) | 13.19 (9.17,18.71) | 19 (13,26) | 0.5 (0.35,0.7) |
| Singapore | 2021 | 493 (409,587) | 5.6 (4.62,6.64) | 21 (17,25) | 0.24 (0.19,0.29) |
| Slovakia | 2021 | 4026 (2787,5623) | 44.63 (30.68,62.35) | 144 (102,199) | 1.54 (1.08,2.12) |
| Slovenia | 2021 | 952 (766,1154) | 24.85 (20.01,30.11) | 35 (28,43) | 0.86 (0.69,1.04) |
| Solomon Islands | 2021 | 47 (33,66) | 12.45 (8.63,17.49) | 2 (1,2) | 0.5 (0.35,0.7) |
| Somalia | 2021 | 1337 (727,2170) | 18.39 (10.25,29.15) | 42 (24,67) | 0.66 (0.37,1.02) |
| South Africa | 2021 | 13328 (11150,15619) | 26.26 (21.97,30.7) | 447 (375,520) | 0.92 (0.78,1.08) |
| South Sudan | 2021 | 783 (462,1205) | 17.6 (10.42,26.88) | 26 (15,40) | 0.67 (0.41,1.01) |
| Spain | 2021 | 25394 (21488,30006) | 29.85 (25.22,35.41) | 1041 (875,1221) | 1.12 (0.95,1.32) |
| Sri Lanka | 2021 | 3337 (1909,5194) | 11.89 (6.89,18.39) | 131 (76,203) | 0.48 (0.29,0.74) |
| Sudan | 2021 | 5771 (3455,8778) | 27.35 (16.87,41.82) | 206 (128,315) | 1.11 (0.7,1.66) |
| Suriname | 2021 | 94 (65,132) | 13.87 (9.65,19.48) | 3 (2,5) | 0.51 (0.35,0.72) |
| Sweden | 2021 | 888 (734,1066) | 4.39 (3.61,5.24) | 42 (35,51) | 0.19 (0.15,0.22) |
| Switzerland | 2021 | 1673 (1440,1906) | 10.23 (8.84,11.65) | 72 (61,82) | 0.4 (0.34,0.45) |
| Syrian Arab Republic | 2021 | 2752 (1907,3888) | 19.29 (13.57,26.9) | 101 (71,142) | 0.82 (0.59,1.11) |
| Taiwan (Province of China) | 2021 | 4990 (4365,5587) | 12 (10.48,13.49) | 193 (169,216) | 0.45 (0.4,0.51) |
| Tajikistan | 2021 | 920 (560,1406) | 13.32 (8.18,20.29) | 30 (18,45) | 0.48 (0.3,0.74) |
| Thailand | 2021 | 25168 (18365,33644) | 22.94 (16.8,30.51) | 971 (703,1292) | 0.88 (0.64,1.17) |
| Timor-Leste | 2021 | 131 (86,197) | 14.95 (9.9,22.42) | 5 (3,8) | 0.62 (0.42,0.89) |
| Togo | 2021 | 839 (578,1156) | 19.37 (13.44,26.57) | 28 (20,39) | 0.73 (0.51,0.99) |
| Tokelau | 2021 | 0 (0,0) | 6.9 (4.71,10) | 0 (0,0) | 0.3 (0.2,0.43) |
| Tonga | 2021 | 7 (5,10) | 8.81 (6.06,12.23) | 0 (0,0) | 0.39 (0.27,0.54) |
| Trinidad and Tobago | 2021 | 368 (267,489) | 18.58 (13.51,24.72) | 14 (10,18) | 0.7 (0.51,0.92) |
| Tunisia | 2021 | 5613 (3784,7934) | 40.49 (27.44,57.18) | 215 (147,300) | 1.62 (1.12,2.25) |
| Türkiye | 2021 | 32429 (24300,42337) | 33.32 (24.97,43.4) | 1260 (951,1635) | 1.34 (1.02,1.74) |
| Turkmenistan | 2021 | 799 (589,1059) | 17.13 (12.66,22.67) | 25 (19,34) | 0.58 (0.43,0.77) |
| Tuvalu | 2021 | 1 (1,1) | 9.7 (7.32,12.92) | 0 (0,0) | 0.4 (0.3,0.52) |
| Uganda | 2021 | 2569 (1702,3971) | 15.5 (10.43,23.45) | 86 (58,130) | 0.58 (0.4,0.85) |
| Ukraine | 2021 | 31345 (19989,45586) | 43.71 (27.78,63.78) | 1055 (674,1528) | 1.41 (0.9,2.05) |
| United Arab Emirates | 2021 | 695 (463,1001) | 13.05 (9.31,17.46) | 20 (14,28) | 0.6 (0.43,0.8) |
| United Kingdom | 2021 | 15102 (13469,16726) | 12.71 (11.37,14.02) | 652 (573,732) | 0.5 (0.44,0.56) |
| United Republic of Tanzania | 2021 | 4329 (2779,7000) | 15.23 (10.04,24.31) | 147 (97,233) | 0.57 (0.38,0.89) |
| United States of America | 2021 | 83635 (74976,91498) | 14.93 (13.43,16.28) | 3279 (2894,3638) | 0.56 (0.49,0.62) |
| United States Virgin Islands | 2021 | 24 (16,35) | 14.1 (9.22,20.05) | 1 (1,1) | 0.54 (0.35,0.78) |
| Uruguay | 2021 | 2391 (2112,2752) | 48.63 (43.05,55.56) | 94 (82,109) | 1.8 (1.58,2.07) |
| Uzbekistan | 2021 | 2877 (2213,3709) | 9.63 (7.41,12.22) | 96 (75,122) | 0.35 (0.28,0.44) |
| Vanuatu | 2021 | 11 (7,16) | 6.27 (4.12,8.77) | 0 (0,1) | 0.27 (0.18,0.36) |
| Venezuela (Bolivarian Republic of) | 2021 | 7863 (5542,10560) | 25.26 (17.84,33.99) | 316 (223,426) | 1.05 (0.75,1.41) |
| Viet Nam | 2021 | 29760 (21095,40377) | 27.41 (19.62,36.61) | 1039 (748,1377) | 1.02 (0.75,1.33) |
| Yemen | 2021 | 6724 (4213,9851) | 43.5 (27.44,62.74) | 234 (147,338) | 1.72 (1.09,2.46) |
| Zambia | 2021 | 1807 (863,4439) | 22.73 (11.4,53.76) | 59 (30,138) | 0.83 (0.44,1.84) |
| Zimbabwe | 2021 | 3344 (2406,4590) | 41.57 (30.44,55.92) | 107 (79,145) | 1.48 (1.12,1.96) |

**Table S2. Mortality, DALYs, and Related Age - standardized Rates of LCAS in 1990: Global and Regional Estimates.**

| Characteristics | Deaths |  | DALYs |  |
| --- | --- | --- | --- | --- |
|  | Counts,2021 | Age-standardized rate per 100 000,2021 | Counts,2021 | Age-standardized rate per 100 000,2021 |
| Global | 64528 (58868,69741) | 1.61 (1.47,1.74) | 1848004 (1688370,1995684) | 44.42 (40.58,47.99) |
| SEX |  |  |  |  |
| Males | 64528 (58868,69741) | 3.3 (3.01,3.56) | 1738738 (1592058,1874986) | 87.92 (80.47,94.8) |
| Females | 4135 (3345,4831) | 0.2 (0.16,0.23) | 109266 (88596,127526) | 5.08 (4.12,5.93) |
| GBD Region |  |  |  |  |
| Andean Latin America | 112 (90,137) | 0.58 (0.47,0.71) | 2730 (2205,3356) | 13.36 (10.78,16.42) |
| Australasia | 208 (185,233) | 0.88 (0.79,0.99) | 5455 (4865,6160) | 23.71 (21.15,26.8) |
| Caribbean | 525 (466,588) | 2.05 (1.82,2.29) | 13428 (11931,14865) | 51.3 (45.61,56.8) |
| Central Asia | 982 (911,1057) | 1.99 (1.84,2.14) | 30369 (28254,32631) | 59.58 (55.42,64) |
| Central Europe | 4611 (4284,4938) | 3.03 (2.81,3.24) | 139647 (130170,149362) | 92.17 (85.92,98.62) |
| Central Latin America | 1040 (937,1136) | 1.34 (1.21,1.47) | 26013 (23554,28314) | 31.06 (28.11,33.86) |
| Central Sub-Saharan Africa | 171 (117,234) | 0.75 (0.53,1.01) | 5060 (3433,6992) | 20.01 (13.79,27.45) |
| East Asia | 10187 (8230,12063) | 1.21 (0.98,1.42) | 282986 (228635,336596) | 30.56 (24.74,36.3) |
| Eastern Europe | 7888 (7450,8315) | 2.75 (2.59,2.9) | 250156 (236016,264173) | 88.11 (83,93.32) |
| Eastern Sub-Saharan Africa | 575 (439,709) | 0.77 (0.59,0.95) | 16759 (12791,20680) | 20.54 (15.68,25.34) |
| High-income Asia Pacific | 1361 (1177,1519) | 0.68 (0.59,0.76) | 34852 (29803,39628) | 16.85 (14.43,19.13) |
| High-income North America | 3713 (3435,3970) | 1.09 (1.01,1.16) | 99099 (92586,105614) | 30.35 (28.41,32.31) |
| North Africa and Middle East | 2834 (2318,3459) | 1.72 (1.41,2.11) | 80264 (65451,97202) | 44.31 (36.12,53.87) |
| Oceania | 10 (7,13) | 0.37 (0.27,0.49) | 279 (196,374) | 8.93 (6.43,11.93) |
| South Asia | 13143 (10767,15687) | 2.28 (1.86,2.71) | 386047 (318977,459765) | 60.95 (50.26,72.68) |
| Southeast Asia | 2410 (2055,2732) | 0.98 (0.83,1.11) | 67171 (57493,76275) | 24.84 (21.25,28.06) |
| Southern Latin America | 1070 (965,1196) | 2.28 (2.05,2.55) | 30925 (27896,34468) | 65.72 (59.28,73.26) |
| Southern Sub-Saharan Africa | 389 (316,513) | 1.42 (1.15,1.88) | 11729 (9523,15292) | 40.11 (32.53,52.54) |
| Tropical Latin America | 2104 (1965,2239) | 2.26 (2.11,2.42) | 61946 (57979,65834) | 62.52 (58.41,66.44) |
| Western Europe | 10815 (10035,11532) | 1.94 (1.81,2.07) | 292190 (272561,311381) | 55.38 (51.75,59.03) |
| Western Sub-Saharan Africa | 380 (295,488) | 0.43 (0.34,0.55) | 10900 (8409,14017) | 11.57 (8.93,14.88) |

**Table S3. Decomposition Analysis of Changes in the Number of Deaths of LCAS in the World and 21 Regions**

| location_name | Overll difference | Aging | Population | Epidemiological change | a_percent | p_percent | r_percent | val_1990 | val_2021 |
| --- | --- | --- | --- | --- | --- | --- | --- | --- | --- |
| Andean Latin America | 50.28 | 28.959 | 134.445 | -113.127 | 57.6 | 267.39 | -224.99 | 111.5029 | 161.7799 |
| Australasia | -61.35 | 41.94 | 118.663 | -221.949 | -68.36 | -193.42 | 361.78 | 207.8531 | 146.5077 |
| Caribbean | 445.31 | 115.427 | 431.721 | -101.837 | 25.92 | 96.95 | -22.87 | 525.3893 | 970.7008 |
| Central Asia | -274.56 | 4.564 | 564.127 | -843.248 | -1.66 | -205.47 | 307.13 | 982.1587 | 707.6017 |
| Central Europe | -533.18 | 655.937 | 632.379 | -1821.49 | -123.02 | -118.61 | 341.63 | 4611.016 | 4077.838 |
| Central Latin America | 111.56 | 362.964 | 1113.69 | -1365.1 | 325.35 | 998.29 | -1223.64 | 1040.143 | 1151.701 |
| Central Sub-Saharan Africa | 154.61 | -24.968 | 247.262 | -67.681 | -16.15 | 159.93 | -43.78 | 170.8697 | 325.4817 |
| East Asia | 5478.71 | 4711.479 | 8477.481 | -7710.25 | 86 | 154.73 | -140.73 | 10186.98 | 15665.7 |
| Eastern Europe | -3063.89 | 446.443 | 630.216 | -4140.55 | -14.57 | -20.57 | 135.14 | 7888.307 | 4824.42 |
| Eastern Sub-Saharan Africa | 273.95 | -70.417 | 681.122 | -336.756 | -25.7 | 248.63 | -122.93 | 574.5134 | 848.4618 |
| High-income Asia Pacific | -182.37 | 749.414 | 475.934 | -1407.72 | -410.93 | -260.97 | 771.9 | 1360.845 | 1178.475 |
| High-income North America | -135.26 | 865.318 | 1574.683 | -2575.26 | -639.74 | -1164.19 | 1903.93 | 3712.568 | 3577.309 |
| North Africa and Middle East | 1950.601025 | 57.579 | 3960.939 | -2067.92 | 2.95 | 203.06 | -106.01 | 2834.176 | 4784.777 |
| Oceania | 9.81 | 0.36 | 13.406 | -3.953 | 3.67 | 136.66 | -40.3 | 9.740833 | 19.55498 |
| South Asia | 9131.045681 | 2596.563 | 14703.99 | -8169.5 | 28.44 | 161.03 | -89.47 | 13143.48 | 22274.52 |
| Southeast Asia | 2601.76 | 703.237 | 2854.449 | -955.93 | 27.03 | 109.71 | -36.74 | 2410.109 | 5011.865 |
| Southern Latin America | -292.48 | 81.427 | 526.885 | -900.787 | -27.84 | -180.14 | 307.98 | 1069.784 | 777.3087 |
| Southern Sub-Saharan Africa | 227.84 | 13.469 | 384.037 | -169.668 | 5.91 | 168.56 | -74.47 | 389.4068 | 617.2457 |
| Tropical Latin America | 1529.39 | 687.977 | 2356.741 | -1515.32 | 44.98 | 154.1 | -99.08 | 2103.907 | 3633.301 |
| Western Europe | -4355.95 | 1334.968 | 2517.013 | -8207.93 | -30.65 | -57.78 | 188.43 | 10815 | 6459.045 |
| Western Sub-Saharan Africa | 357.41 | -66.786 | 517.831 | -93.638 | -18.69 | 144.88 | -26.2 | 380.029 | 737.4358 |
| Global | 13423.25 | 10935.11 | 48142.23 | -45654.1 | 81.46 | 358.65 | -340.11 | 64527.78 | 77951.03 |

**Table S4. Decomposition Analysis of Changes in the Number of DALYs of LCAS in the World and 21 Regions**

| location_name | Overll difference | Aging | Population | Epidemiological change | a_percent | p_percent | r_percent | val_1990 | val_2021 |
| --- | --- | --- | --- | --- | --- | --- | --- | --- | --- |
| Andean Latin America | 1011.16 | 564.065 | 3212.252 | -2765.156 | 55.78 | 317.68 | -273.46 | 2729.516 | 3740.676 |
| Australasia | -1997.88 | 865.882 | 3016.638 | -5880.4 | -43.34 | -150.99 | 294.33 | 5455.197 | 3457.317 |
| Caribbean | 11376.48 | 2604.8 | 11012.056 | -2240.374 | 22.9 | 96.8 | -19.69 | 13428.03 | 24804.51 |
| Central Asia | -9764.54 | 666.06 | 17189.16 | -27619.763 | -6.82 | -176.04 | 282.86 | 30368.61 | 20604.07 |
| Central Europe | -29735.39 | 11250.482 | 18178.793 | -59164.665 | -37.84 | -61.14 | 198.97 | 139646.8 | 109911.4 |
| Central Latin America | 1296.58 | 7478.131 | 27171.341 | -33352.889 | 576.76 | 2095.62 | -2572.37 | 26012.72 | 27309.3 |
| Central Sub-Saharan Africa | 4787.13 | -605.566 | 7393.297 | -2000.603 | -12.65 | 154.44 | -41.79 | 5059.526 | 9846.655 |
| East Asia | 109280.11 | 97999.776 | 224095.138 | -212814.808 | 89.68 | 205.06 | -194.74 | 282985.6 | 392265.7 |
| Eastern Europe | -110906.85 | 7516.835 | 19295.63 | -137719.317 | -6.78 | -17.4 | 124.18 | 250156.4 | 139249.5 |
| Eastern Sub-Saharan Africa | 8488.26 | -1763.56 | 20038.062 | -9786.244 | -20.78 | 236.07 | -115.29 | 16759.25 | 25247.51 |
| High-income Asia Pacific | -11567.97 | 11165.089 | 10840.794 | -33573.854 | -96.52 | -93.71 | 290.23 | 34851.86 | 23283.89 |
| High-income North America | -8506.18 | 22036.556 | 41207.617 | -71750.357 | -259.07 | -484.44 | 843.51 | 99099.2 | 90593.02 |
| North Africa and Middle East | 100175.47 | 50087.735 | 1199.5015 | 110578.8185 | 2.39 | 220.77 | -123.17 | 80263.76 | 130351.5 |
| Oceania | 278.23 | 8.533 | 382.955 | -113.255 | 3.07 | 137.64 | -40.71 | 278.9067 | 557.1399 |
| South Asia | 447566.07 | 223783.035 | 51208.173 | 418410.322 | 22.88 | 186.97 | -109.85 | 386046.6 | 609829.6 |
| Southeast Asia | 68772.29 | 17429.108 | 78480.555 | -27137.373 | 25.34 | 114.12 | -39.46 | 67170.62 | 135942.9 |
| Southern Latin America | -10840.79 | 1509.126 | 14704.682 | -27054.603 | -13.92 | -135.64 | 249.56 | 30925.21 | 20084.42 |
| Southern Sub-Saharan Africa | 6810.97 | 523.166 | 11560.887 | -5273.079 | 7.68 | 169.74 | -77.42 | 11729.02 | 18540 |
| Tropical Latin America | 37413.51 | 15616.547 | 66995.689 | -45198.728 | 41.74 | 179.07 | -120.81 | 61945.95 | 99359.46 |
| Western Europe | -137425.51 | 23042.046 | 65270.373 | -225737.928 | -16.77 | -47.5 | 164.26 | 292190.5 | 154764.9 |
| Western Sub-Saharan Africa | 10469.12 | -1441.349 | 14926.227 | -3015.76 | -13.77 | 142.57 | -28.81 | 10900.36 | 21369.48 |
| Global | 213109.49 | 238070.57 | 1335365.24 | -1360326.325 | 111.71 | 626.61 | -638.32 | 1848004 | 2061113 |
